# FPGA-based emulation framework for two-qubit quantum computation: Design, implementation, and validation

**DOI:** 10.1371/journal.pone.0356172

**Published:** 2026-08-24

**Authors:** Arfeen Zahra, Madiha Khalid, Naveed Riaz, Salman Waheed, Umar Mujahid, Muhammad Najam-ul-Islam

**Affiliations:** 1 Department of Computing, School of Electrical Engineering and Computer Science, National University of Sciences and Technology (NUST), Islamabad, Pakistan; 2 Department of Electrical and Computer Engineering, International Islamic University, Islamabad, Pakistan; 3 Department of Information Technology, Georgia Gwinnett College, Lawrenceville, Georgia, United States of America; 4 Department of Electrical Engineering, Namal University, Mianwali, Pakistan; Commonwealth Scientific and Industrial Research Organisation, AUSTRALIA

## Abstract

Quantum computing leverages quantum mechanics to perform complex computations efficiently, yet its practical implementation is hindered by hardware-software interdependence and quantum state instability. This paper advances lightweight FPGA-based quantum emulation by incorporating a universal quantum gate set, including parameterized *Phase* gates and the CNOT gate, to support comprehensive quantum protocol emulation on FPGA hardware. The FPGA platform used for synthesis is *Artix-7*. The proposed emulator is validated through simulations of quantum circuits, including Bell States, the Deutsch algorithm, Superdense coding, Grover’s search, and BB84 QKD, with results benchmarked against IBM’s noiseless quantum simulator. Utilizing FPGA’s customizable parallelism and energy efficiency, this framework offers a resource-efficient, reliable platform for developing and testing quantum algorithms.

## 1 Introduction

In 1980, Richard Feynman and Paul Benioff presented the idea of a Quantum Computer, i.e., a hardware system based on the principles of quantum mechanics. Unlike classical computers, which operate on binary logic, this technology leverages the laws of quantum physics, allowing the system to perform powerful operations and execute certain computations efficiently.

In the present information era, Quantum Information Technology (QIT) is a field of interest for businesses, investors, and nations, as it offers increased computational power and precision in various application areas, including sensing, information security, drug discovery, optimization, and telecommunication. Core disciplines in Quantum Computing (QC) are quantum platform and algorithm design. Theoretical work on quantum algorithm design began in the latter half of the 20${\;}^{th}$ century with algorithms such as Deutsch’s, Shor’s, and Grover’s, and has since evolved to include concepts like variational quantum eigensolvers, quantum approximate optimization, and Quantum Machine Learning (QML) [6–8]. While quantum algorithms have matured over time, the creation of practical execution platforms has historically been constrained by the complex interdependence of hardware and software, as well as the fundamental challenge of maintaining stable quantum states. Over the past decade, however, technological advances have enabled the construction of devices capable of deliberately manipulating nature at its fundamental quantum-mechanical level. These advances in quantum hardware have, in turn, motivated renewed interest in near-term algorithmic applications. The technologies used in the development of quantum computing hardware architecture are summarized in Table 1. Since quantum computing is both capital-intensive and strategically important, access to this technology is primarily restricted to multinational enterprises and technologically advanced economies. Organizations such as Google, Amazon, and D-Wave are at the forefront of investment. Currently, the most extensive system is reported by IBM, featuring 1,121 qubits, i.e., the quantum analog of a classical bit [9,10].

**Table 1:** Quantum computing technologies and their qubit representing entities.

S. No	Technology	Qubit-representing entity
1	Photonics Networks	Occupation of a photonic waveguide [1]
2	Linear-optics quantum computer	The optical mode of the photon [2]
3	Superconducting Circuits	Superconducting qubits [3]
4	Spin Qubits	Electron spins (laser light) [4]
5	Ion-trapped-based computer	Quantized ion states [5]

This limitation has given rise to a parallel line of research focused on emulating quantum computing using classical devices. The primary advantage of this approach lies in providing a controlled environment for exploring quantum systems, thereby mitigating the challenges of noise, decoherence, and high error rates. However, this comes at the expense of an exponential increase in computational resources with each additional qubit. These emulation platforms enable reliable, repeatable simulations, allowing for effective development, testing, and refinement of quantum algorithms before they are executed on actual hardware.

### 1.1 Motivation

Quantum emulators have been developed on various platforms, including Graphics Processing Units (GPUs), Central Processing Units (CPUs), and Field-Programmable Gate Arrays (FPGAs), providing platforms for executing quantum algorithms. In contrast to CPUs and GPUs, FPGAs offer the potential of customizable parallelism, energy efficiency, and flexible hardware configuration [11], making them an enabling technology for a resource-efficient yet accurate quantum emulator.

In 2021, Khalid et al. introduced a generalized, lightweight FPGA-based emulation architecture capable of simulating quantum superposition [12]. The proposed framework was synthesized on *Spartan-3*, keeping in view its focus on low computational cost. Given the accuracy of the BB84 emulation results demonstrated in that work, extending the framework to incorporate the full set of primary quantum gates, particularly the CNOT gate, is a notable research direction.

### 1.2 Contributions

The list of contributions presented in the paper is as follows:

1. Extension of the reference FPGA-based quantum emulation framework by completing the universal set of quantum gates through the proposed *Phase* and CNOT gate architectures.
2. Validation of the proposed emulator through the simulation of representative quantum circuits, including Bell States, the Deutsch algorithm, Grover’s Search, BB84 QKD, and the parallel execution of multiple independent 2-qubit Superdense Coding circuits to demonstrate the scalability of the proposed framework.
3. Functional benchmarking of the proposed emulator against IBM Aer Simulator and IBM Quantum Hardware.

The proposed architectures are designed on *Artix-7* in contrast to *Spartan-3*, i.e., the platform used by the baseline framework. This board is chosen to provide the required logic density and on-chip memory needed to instantiate and manage the emulated quantum circuits within the Finite State Machine (FSM) driven model.

### 1.3 Paper organization

This paper is structured as follows: Section 2 comprehensively reviews related work, exploring existing quantum simulation frameworks and their limitations to contextualize the proposed solution. Section 3 details the proposed FPGA emulator’s system architecture, while section 4 describes the implementation and theoretical benchmarking of quantum algorithms, validating their execution against expected outcomes. Section 5 presents the performance analysis, evaluating the emulator’s efficacy compared to other platforms, i.e., the IBM Quantum platform. Finally, the paper is concluded in Section 6.

## 2 Related work

The related work on QC emulation can be broadly categorized into two dimensions:

1. The mathematical fundamentals underlying quantum phenomena.
2. The existing literature on emulation architectures and design.

The subsequent subsections address these topics in sequence.

### 2.1 Fundamentals of quantum computing

Quantum analog of a classical bit is a qubit. While classical computers use the transistor theory to represent a binary bit, QC utilizes nanoparticles to hold information in the form of *spin*, i.e., an intrinsic quantum property of elementary particles. The mathematical abstraction of this physical property is a unit vector |ψ⟩ expressed as a linear combination of two orthogonal basis states in a two-dimensional complex Hilbert space. Mathematical representation of a qubit, i.e., an elementary particle *spin*, is expressed in Eq 1.


$$\begin{array}{c} |\psi \rangle =p_{0}\left|↑\right\rangle +p_{1}\left|↓\right\rangle \\ |p_{0}{|}^{2}+|p_{1}{|}^{2}=1 \end{array}$$ (1)

where *p*_0_ and *p*_1_ are complex numbers that define the projections of |ψ⟩ on the basis states |↑⟩ and |↓⟩.

Since complex amplitudes represent quantum states, quantum computation relies on the algebra of complex numbers. To establish the foundation, the representation of complex variables in both rectangular and polar forms, along with their arithmetic, is presented in Eqs 2–4 respectively.


$$\begin{array}{c} Z=re^{i\theta }=rcos\theta +irsin\theta =a+ib \end{array}$$ (2)

$$\begin{array}{c} \left(a+ib)+(c+id)=(a+c)+i(b+d\right) \end{array}$$ (3)

$$\begin{array}{c} \left(a+ib)(c+id)=(ac-bd)+i(ad+bc\right) \end{array}$$ (4)

These equations provide the mathematical foundation for describing quantum states, their transformations, and measurements.

**Fig 1:**
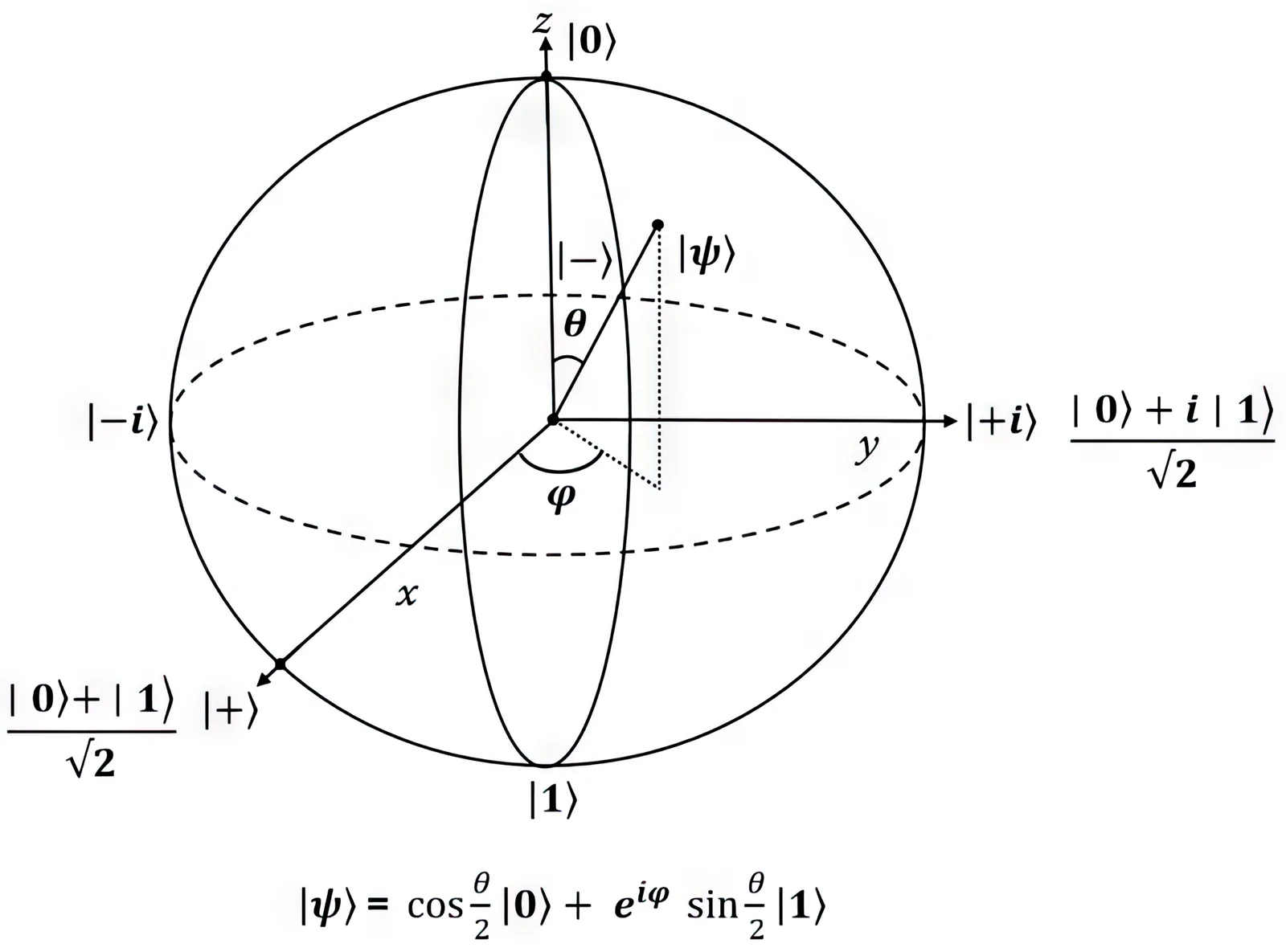
Graphical representation of a qubit: the Bloch sphere.

Graphically, a qubit is represented on a unit sphere known as the Bloch sphere. In this model, the state of a qubit corresponds to a unit vector defined with respect to an orthonormal basis. The standard choice of basis is the computational basis, also known as the Z−basis, denoted as |0⟩ and |1⟩, placed at the north and south poles of the sphere, respectively. Any pure qubit state can then be expressed as a linear combination of these basis states and visualized as a point on the Bloch sphere, as shown in Fig 1.

The core concept of *spin* enables QC with superposition and entanglement. The following is the description of these properties.

1. *Superposition:* states that a qubit can exist in a combination of basis states simultaneously, defined by complex probability amplitudes whose squared magnitudes give the measurement probabilities, unlike a classical bit, which deterministically represents either 0 or 1. The mathematical expression for superposition is defined as Eq 5.
$$\begin{array}{c} |\psi \rangle =C_{0}\left|0\right\rangle +C_{1}\left|1\right\rangle ,\;C_{i}=\alpha_{i}+\hat{ı}\beta_{i},\;\;\text{for }i=0,1 \end{array}$$ (5)
$$\begin{array}{c} P(i)=|C_{i}{|}^{2},\;\text{where }i\in \{0,1\} \end{array}$$ (6)
  The probability states, i.e., $C_{i}$ is a complex number defined by a combination of real ($\alpha_{i}$) and imaginary ($\beta_{i}$) parts. In Eq 6, *P*(.) denotes the probability of a measurement event.
  The basis states (|0⟩,|1⟩) are represented as a column vector of dimension 2×1, expressed in Dirac notation as a *ket*, as shown in Eq 7.
$$\begin{array}{c} \left|\psi \rangle =[\begin{array}{c} C_{0}\\ C_{1} \end{array}]=C_{0}[\begin{array}{c} 1\\ 0 \end{array}]+C_{1}[\begin{array}{c} 0\\ 1 \end{array}];\;|0\rangle \to [\begin{array}{c} 1\\ 0 \end{array}],\;|1\rangle \to [\begin{array}{c} 0\\ 1 \end{array}\right] \end{array}$$ (7)
2. *Entanglement:* It is a phenomenon where two or more qubits are linked, such that their quantum states cannot be described independently, even when separated by vast distances. Measuring the state of one entangled particle instantly determines the state of the other. Mathematically, the entangled qubits are represented as non-separable states in the tensor product space in Eq 8.
$$\begin{array}{cc} |\eta \rangle & =C_{00}|00\rangle +C_{01}|01\rangle +C_{10}|10\rangle +C_{11}|11\rangle \\ {}[2mm]C_{ij} & =\alpha_{ij}+\hat{ı}\;\beta_{ij},\;P_{ij}=|C_{ij}{|}^{2},\;\text{where }i,j\in \{0,1\} \end{array}$$ (8)

**Fig 2:**
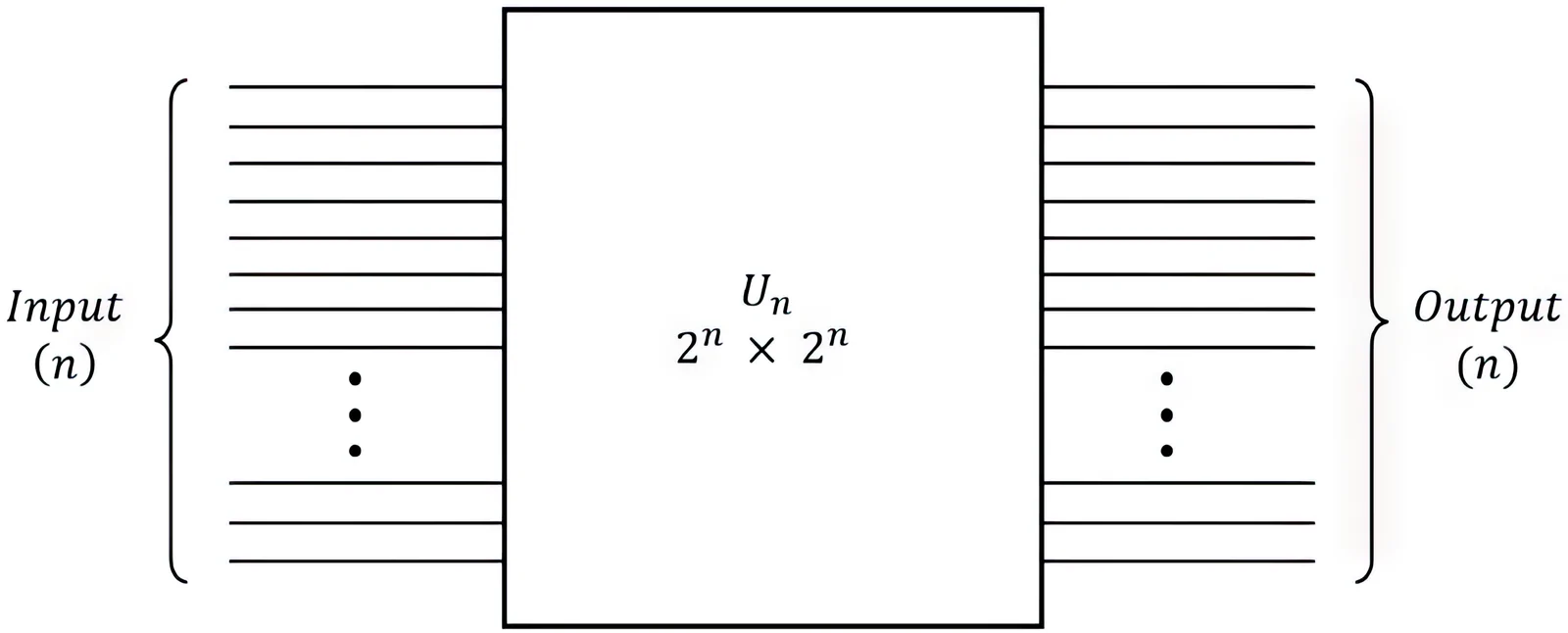
*n*-qubit quantum circuit.

In the circuit model of quantum computation, *wires* carry logical qubits and quantum gates act on qubits, i.e., gates perform reversible phase shifts on the operand qubits. A quantum gate acting on *n* qubits has *n* wires as input and output, and a $2^{n}\times 2^{n}$ unitary matrix represents the gate. Figure 2 represents an *n*-qubit quantum circuit.

A quantum algorithm can be abstracted as a complex unitary operator acting on *n* qubits. In practice, this complex operator is broken down into a sequence of operations from a universal set of quantum gates, i.e., a set composed of 2-qubit entangling gates, such as CNOT, and all 1-qubit gates. Since P(θ) provides arbitrary *Z*-axis rotation and Hadamard conjugates *Z* into *X*, the pair [H,P(θ)] generates arbitrary single qubit unitary operations via Euler angle decomposition. Together with CNOT, which is an entangling 2-qubit gate, the set [H,P(θ),CNOT] is universal for quantum computations [13].

Table 2 presents the rudimentary set of universal gates. CNOT gate takes *n* = 2 inputs, i.e., a control qubit $\left|q_{0}\right\rangle$ and a target qubit $\left|q_{1}\right\rangle$. The state of the target qubit alters based on the control qubit, resulting in entanglement.

**Table 2:** Set of quantum computing unitary gates.

Single qubit gates		
**Gate**	**Matrix Representation**	**Description**
Pauli-X	$\left[\begin{array}{cc} 0 & 1\\ 1 & 0 \end{array}\right]$	Phase-flip along the *x*-axis
Pauli-Y	$\left[\begin{array}{cc} 0 & -i\\ i & 0 \end{array}\right]$	Phase-flip along the *y*-axis
Pauli-Z	$\left[\begin{array}{cc} 1 & 0\\ 0 & -1 \end{array}\right]$	Phase-flip along the *z*-axis
Hadamard	$\frac{1}{\sqrt{2}}[\begin{array}{cc} 1 & 1\\ 1 & -1 \end{array}]$	π/2 rotation about the *y*-axis followed by a π rotation about the *x*-axis
Phase Gate-P(θ)	$\left[\begin{array}{cc} 1 & 0\\ 0 & e^{i\theta } \end{array}\right]$	Rotation of the phase by angle θ along the *z*-axis
**Two qubit gate**		
**Gate**	**Matrix Representation**	**Description**
Controlled-NOT (CNOT, CX)	$\left[\begin{array}{cccc} 1 & 0 & 0 & 0\\ 0 & 1 & 0 & 0\\ 0 & 0 & 0 & 1\\ 0 & 0 & 1 & 0 \end{array}\right]$	Two-qubit entangling gate

### 2.2 Literature review on quantum emulations

The mathematics behind quantum information processing shows that a quantum algorithm can be represented as a series of unitary transformations, simplifying the quantum computing solution to matrix multiplications. The literature review indicates that emulation and simulation setups execute the state space representation of quantum circuits through matrix-based operations.

Over the past decade, FPGA-based quantum emulation has progressed from basic gate-level models to more sophisticated algorithmic abstractions. However, scalability and efficiency remain a persistent challenge. To improve gate accuracy, Giorgio et al. [14] proposed model-based design and optimized HDL, though fixed-point arithmetic still restricts scalability. Recent work by Conti et al. [15] and Lagostina et al. [16] focused on improving resource efficiency and, consequently, enhancing scalability. Despite these advances, hardware limitations continue to restrict both efficiency and scalability.

In addition to advances in FPGA-based quantum emulation, parallel research focuses on accelerating specific quantum algorithms, such as the Quantum Fourier Transform (QFT), Haar Transform, and Grover’s algorithm. Nedjah et al. [17] achieved speedups of 1.7 ns vs. 29.6 μs for two qubits by using dedicated FPGA hardware, but support was limited to six qubits. Waidyasooriya et al. [18] implemented a 30-qubit QFT emulator, achieving 23.6x-24.5x speedup over CPU with high fidelity, but resource and synchronization challenges remained. El-Araby et al. [19] emulated up to 32 qubits with a 21.66x speedup for *C*2*Q* and a 3.49x speedup for QHT compared to CPU, along with a 25% reduction in circuit depth, though hardware demands quickly became prohibitive. Choi et al. [20] and Mahmud et al. [21] advanced Grover’s and QFT emulation, and achieved 6-qubit emulation on Kintex (vs. 4-qubit on Arty A7), with 191x performance boost over software. Still, approximation errors up to 6.25 × 10^−4^, scaling challenges with increasing qubit count, and exponential memory growth continued to limit large-scale implementations. In the comparative analysis, the architectures [16] and [21] indicate quantum emulation but do not explicitly specify the number of qubits. In such architectures, the number of representable qubits primarily depends on the register size and the allocation of available hardware resources.

Collectively, these works demonstrate the feasibility and potential of FPGA-based emulation. They highlight the need to improve hardware and design strategies to create an optimized, lightweight solution for scalable, general-purpose applications and to support larger, more complex quantum systems. Table 3 summarises the key hardware abstraction circuits presented in the literature, organised by gate-level and algorithmic-level approaches.

**Table 3:** Comparative analysis of FPGA-based quantum emulation approaches.

Emulation Approach	Benchmarking	#Qubits	Precision	Platform
**Gate Level Abstraction**				
Lagostina et al. [16], 2025	X/Y/Z/H/S/S${\;}^{†}$/T/T${\;}^{†}$, RX, RY, RZ, CNOT, QFT	–	20-bit fixed-point	Altera Cyclone 10LP FPGA
Giorgio et al. [14], 2024	Single-Gates/CNOT	2	16-bit fixed-point	Cyclone V/Arria10 FPGA
Conti et al. [15], 2024	Clifford+T/Rotational gate sets	16	20-bit fixed-point	AMD Kria KV260 SoM FPGA
Khalid et al. [12], 2021	Pauli−X/Y/Z, *H*, BB84 (QKD)	8	8-bit fixed-point	FPGA (ISE)
**Quantum Algorithms Specific**				
Nedjah et al. [17], 2024	Grover’s search	6	32-bit floating-point	Xilinx Virtex UltraScale
Choi et al. [20], 2024	Grover’s Search	4−6	32-bit fixed-point	Kintex UltraScale + , Artix-7
El-Araby et al. [19], 2023	QHT	32	32-bit floating-point	HPRC
Waidyasooriya et al. [18], 2022	QFT	30	floating-point	Intel Stratix 10 MX FPGA
Mahmud et al. [21], 2020	QFT, Grover’s search	–	fixed-point	Intel–Altera Arria 10 FPGA

### 2.3 Baseline framework

Emulations are valuable for evaluating and developing quantum algorithms. These frameworks are inherently memory- and compute-bound, and scalability is ensured only by trading one for the other [12]. In 2021, Khalid et al. presented a *Spartan-3*-based abstraction of a noiseless single-qubit quantum system. The present work is an algorithm-level state-vector emulation that accelerates computation by preprocessing circuit matrices.

In the baseline framework, a qubit |ψ⟩ is represented in the form of probability states as per the expression given in Eq 5. Each probability state is represented as a pair of signed fixed-point numbers. Each probability amplitude is represented using signed (*m* + 2) bits fixed-point forma, i.e., *Q*(2,*m*), consisting of one sign bit, one integer bit, and *m* fractional bits. Although this format provides a dynamic range of [−2, 2–2^-*m*^], the effective range utilized in the proposed architecture is restricted to [−1,1], consistent with the normalization constraint of quantum states. Since probability amplitudes satisfy $|C_{i}|\leq 1$, the additional integer range is not exercised during operation. The resolution of the representation is 2^-*m*^, i.e., the maximum quantization error per component. Increasing *m* therefore reduces amplitude quantization error and correspondingly decreases deviation from the ideal Bloch-sphere representation.

Collectively, a single qubit is given as Eq 9.


$$\begin{array}{c} \left|\psi \right\rangle =[\begin{array}{c} \alpha_{0}+i\beta_{0}\\ \alpha_{1}+i\beta_{1} \end{array}] \end{array}$$ (9)

The FPGA memory representation of this matrix is a register file that stores four signed fixed-point numbers.

The setup demonstrates superposition and probabilistic measurement through single-qubit gates, i.e., Pauli-X, Pauli-Y, Pauli-Z, Hadamard, and measurement. An algorithmic simulation of Quantum Key Distribution (QKD) protocols, i.e., BB84 and B92, verifies the emulation framework by benchmarking the results against theoretical models [22]. Given the *Q*(2,6) fixed-point representation, the qubit emulation is termed as 8-bit state space representation in the base paper’s nomenclature. The baseline framework performs performance analysis on 8, 32, and 64-bit representations, and the results show that this FPGA-based architecture yields highly accurate results when a 64-bit state space represents the circuit.

Given the configurable nature of FPGAs relative to CPUs and GPUs, the control on accuracy achieved by emulation is promising. This highlights the need to extend the base framework by developing all gates in the universal set, including all single-qubit transformations and an entangling gate, such as the CNOT. Therefore, this paper is an extension of the base framework *Artix-7* to include the following gates:

1. Single-qubit gates, i.e., Phase, *S* and *T* gate.
2. 2-qubit gate, i.e., CNOT transformation.

The subsequent sections present the extended architecture of the base framework, along with the associated experimental verifications.

## 3 System architecture of the extended FPGA emulator

**Fig 3:**
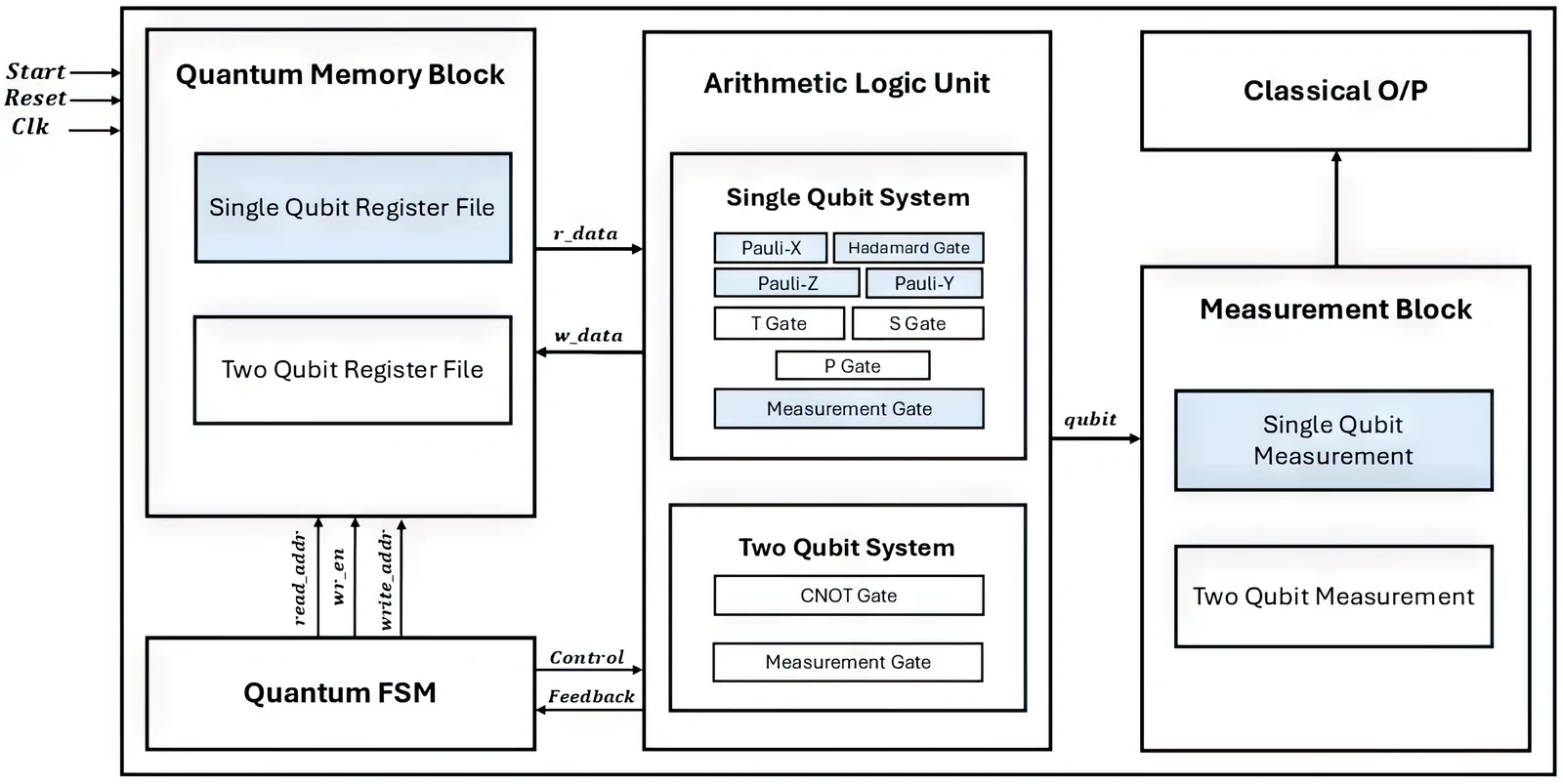
Block diagram of the emulator architecture.

The architecture of the proposed framework is designed to be hierarchical. The Finite State Machine (FSM) is the control module of the emulation that fetches the state vector of quantum information from the memory, transforms it using gates defined in the Arithmetic Logic Unit (ALU), and the result is then stored in quantum memory and can be exported to the measurement block for the classical output. The functional blocks, i.e., ALU, emulated memory, and measurement are composed of two subblocks: a single-qubit system and a 2-qubit system, to support a set of universal gates. Each quantum operation is defined as an instantiable module to ensure reusability and to reduce dependency among functional blocks. The block diagram of the system architecture is presented in Fig 3.

The proposed architecture adopts the same fixed-point representation model as the baseline framework without modification, ensuring direct comparability of numerical precision and performance. Each probability state is represented by two *Q*(2,6) signed numbers, i.e., to represent the real (α) and imaginary (β) parts of a complex state (*C*). The memory footprint of a single qubit is two probability states ($C_{0},C_{1}$) as per Eq 5 stored as four *Q*(2,6) numbers in a register file. Similarly, 2-qubit representation requires four states, i.e., $C_{00},C_{01},C_{10},C_{11}$ as defined in Eq 7 and represented by eight *Q*(2,6) registers stored in a register file.

In addition to this, the quantum operations are unitary, and qubit transformations through these gates are mathematically represented as matrix multiplication. Since the proposed emulation focuses on a lightweight architecture, the matrix multiplications are simplified to optimize the ALU’s computational resources. The details of quantum gate architectures and resource utilization summaries are presented in subsequent subsections. The implementation, including the Verilog source code, experimental workflow, and supporting files, is publicly available on GitHub [23,24].

For the cost-versus-accuracy analysis, the emulation architectures are scaled up to a 32-bit(*Q*(2,30)) representation. The detailed analysis of scaled-up emulation is presented in Section 5. The proposed quantum emulator is designed on Vivado Design Suite using Verilog, and the results are synthesized on *Artix-7*. The resource summary of the target board is given in Table 4.

**Table 4:** On-Chip resources on Xilinx *Artix-7* (xc7a200tfbg676-2).

Resource	Available
LUTs	133,800
Flip-Flops (FFs)	267,600
DSP Blocks	740
I/O Pins	400
BRAMs	365 (36 KB each)

### 3.1 Quantum memory block

In the standard Schrodinger’s approach, the superposition state vector represents an *n*-qubit system by $2^{n}$-dimensional Hilbert space where n∈{1,2}. In the baseline, i.e., *n* = 1 framework, a qubit, mathematically represented as Eq 5, is defined as a 2^1^-dimensional complex Hilbert space and implemented as a register file consisting of four independent *Q*(2,6) signed registers. First two registers define $C_{0}=\alpha_{0}+i\beta_{0}$ by storing $\alpha_{0}$ and $\beta_{0}$ at address 00_2_ and 01_2_ respectively. *C*_1_ is stored similarly in the subsequent registers. The *Q*(2,6) signed fixed-point register file enables the quantum memory to accurately represent the computational basis at the cost of over-provisioning for qubit representation.

This work proposes a *n* = 2 quantum information representation through 2^2^-dimensional complex Hilbert space. The mathematical description of the state vectors is given in Eq 10.


$$\begin{array}{c} \left|\psi \rangle ⊗|\phi \rangle =[\begin{array}{c} C_{\psi 0}\\ {}[6pt]C_{\psi 1} \end{array}]⊗[\begin{array}{c} C_{\phi 0}\\ {}[6pt]C_{\phi 1} \end{array}\right] \end{array}$$ (10)

$$\begin{array}{ccc} & = & \left(C_{\psi 0}|0\rangle +C_{\psi 1}|1\rangle )⊗(C_{\phi 0}|0\rangle +C_{\phi 1}|1\rangle \right) \end{array}$$ (11)

$$\begin{array}{ccc} & = & C_{\psi 0}C_{\phi 0}|00\rangle +C_{\psi 0}C_{\phi 1}|01\rangle +C_{\psi 1}C_{\phi 0}|10\rangle +C_{\psi 1}C_{\phi 1}|11\rangle \end{array}$$ (12)

$$\begin{array}{ccc} & = & C_{00}|00\rangle +C_{01}|01\rangle +C_{10}|10\rangle +C_{11}|11\rangle =[\begin{array}{c} C_{00}\\ C_{01}\\ C_{10}\\ C_{11} \end{array}] \end{array}$$ (13)

To represent four complex numbers of the form C=α+iβ, a register file of structure $2^{3}\times 8$ is used to represent a 2-qubit system.

**Fig 4:**
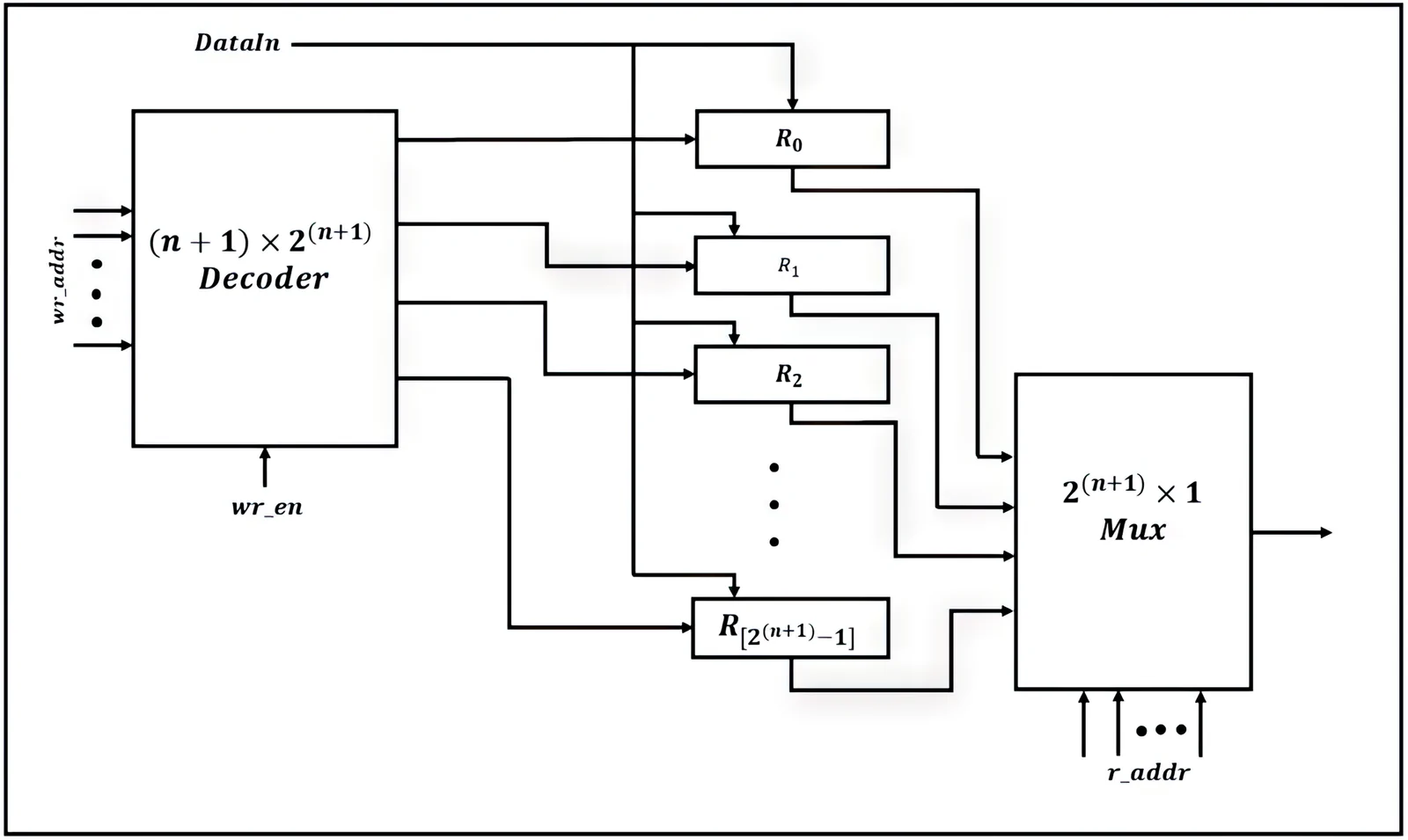
Block diagram of the *n* qubit memory architecture (n∈{1,2}).

Figure 4 presents the memory architecture to store *n*-qubits, where n∈{1,2}. The *write* flag enables the $(n+1)\times 2^{n+1}$ *Decoder* and $2^{n+1}\times 1$ *Mux* for read and write operations at binary values 1 and 0 respectively. The register file acts as an *n*-qubit flip-flop, i.e., the complete file is read sequentially to access the state vectors of quantum information.

### 3.2 Measurement block

The base paper’s measurement architectures employ multi-shot sampling to convert quantum information into classical bits. This process refers to repeated probabilistic measurements of an identically prepared quantum state over *N* trials, in which empirical outcome frequencies approximate the theoretical probabilities.

In a single measurement instance, a Permutation-based Shuffling (PbS) function generates a fixed-point pseudo-random number (PRNG) in the interval [0, 1]. If the PRNG lies within the interval [0, ${\left|C_{0}\right|}^{2}$], the outcome is recorded as |0⟩; otherwise the qubit is measured as |1⟩ [12]. This procedure emulates the stochastic nature of quantum measurement, in which the squared magnitudes of the probability amplitudes determine the likelihood of each outcome.

The proposed upgrade of this block involves the inclusion of a two-qubit measurement to facilitate the calculation of the classical output of the CNOT gate. The architecture is based on a probabilistic sampling principle.

The general state of a 2-qubit system is represented as Eq 14 with the normalised condition on amplitudes.


$$\begin{array}{c} |\eta \rangle =C_{00}|00\rangle +C_{01}|01\rangle +C_{10}|10\rangle +C_{11}|11\rangle ,\;\sum_{i,j\in \{0,1\}}|C_{ij}{|}^{2}=1. \end{array}$$ (14)

For measurement, the interval [0,1] is divided into four subintervals according to the probability distribution of basis states, as elaborated in Eq 15.


$$\left[0,|C_{00}{|}^{2}),\;[|C_{00}{|}^{2},|C_{00}{|}^{2}+|C_{01}{|}^{2}),\;[|C_{00}{|}^{2}+|C_{01}{|}^{2},|C_{00}{|}^{2}+|C_{01}{|}^{2}+|C_{10}{|}^{2}),\;[\cdot ,1\right]$$ (15)

**Fig 5:**
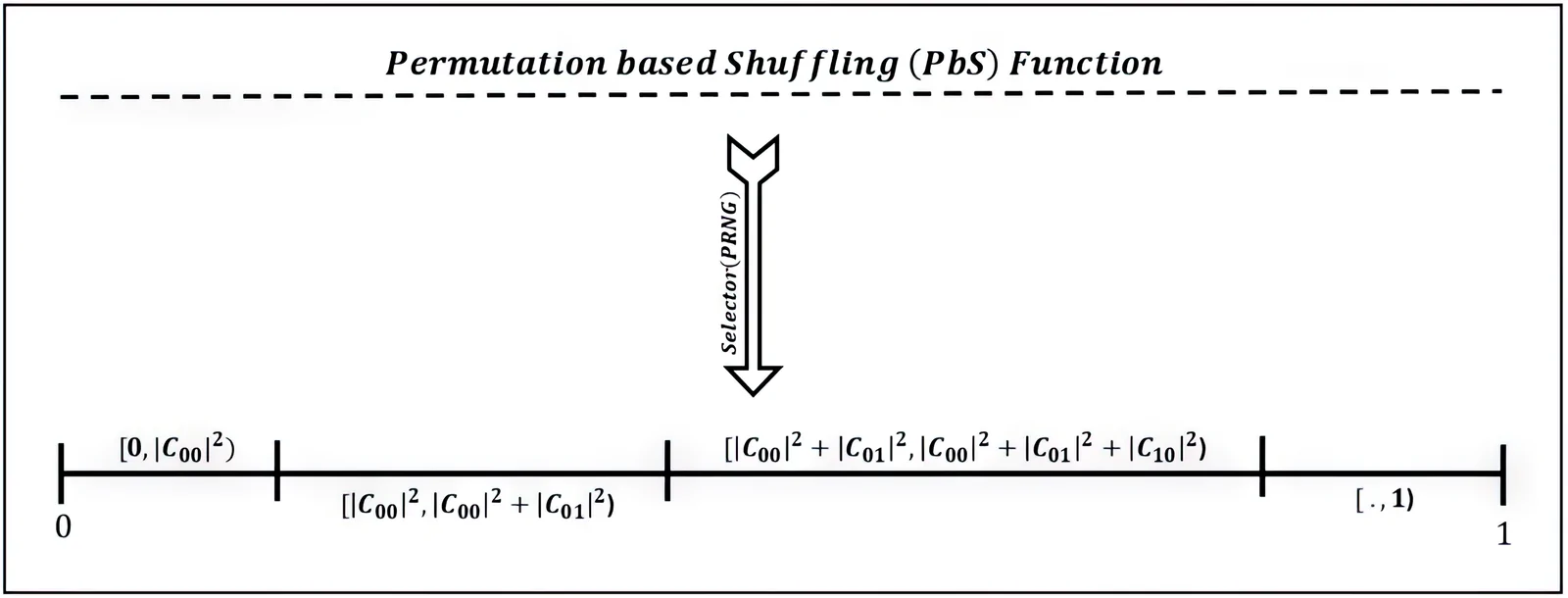
Block diagram of the 2-qubit quantum measurement.

Each subinterval corresponds to the states |00⟩, |01⟩, |10⟩, |11⟩ respectively. For each measurement instance, PbS function generates a PRNG. The measurement outcome is then determined as the basis state whose probability interval contains PRNG. Figure 5 is the abstract level representation of 2-qubit measurement emulation.

In the proposed architecture, each measurement experiment defined in section 5 is executed over 100 shots to implement multi-shot sampling. The resulting empirical frequency distribution converges to the theoretical probabilities defined by the Born rule. The selection of 100 shots provides a balance between statistical reliability and computational efficiency. Furthermore, this configuration ensures methodological consistency with the IBM quantum platform, which similarly reports measurement outcomes using repeated-shot sampling.

### 3.3 Arithmetic logic unit

The ALU is the computational module of the emulation framework that performs quantum state transformation through a sequence of gates scheduled by the FSM and defined by a quantum circuit, i.e., a standard mathematical model to describe computations. Figure 2 presents a generalised quantum circuit that transforms *n*-qubits through a $2^{n}\times 2^{n}$ unitary gate.

This unitary gate can be further simplified to a sequence of unitary gates, i.e., a finite set of elementary gates capable of constructing non-trivial quantum computations. Formally, a set of gates is said to be universal if it is composed of all single-qubit gates and a 2-qubit entangling gate [13]. The base framework partially emulates the universal set, i.e., Pauli-X, Pauli-Y, Pauli-Z, and Hadamard gates. Whereas the proposed work incorporates the remaining single-qubit gates and an entangling 2-qubit gate, completing the set defined in Table 2. The emulation architectures of the designed gates are as follows:

#### 3.3.1 Single-qubit phase gate emulation.

Single-qubit transformation reflects the rotation of the corresponding state vector on the Bloch sphere. Discrete unitary operators are Pauli gates, as these gates apply a π rotation over the reference axis. On the other hand, *Phase* gates use finer control over azimuthal rotation on the Bloch sphere, i.e., a continuous rotation of the state vector by angle θ along the *z*-axis.

**Fig 6:**
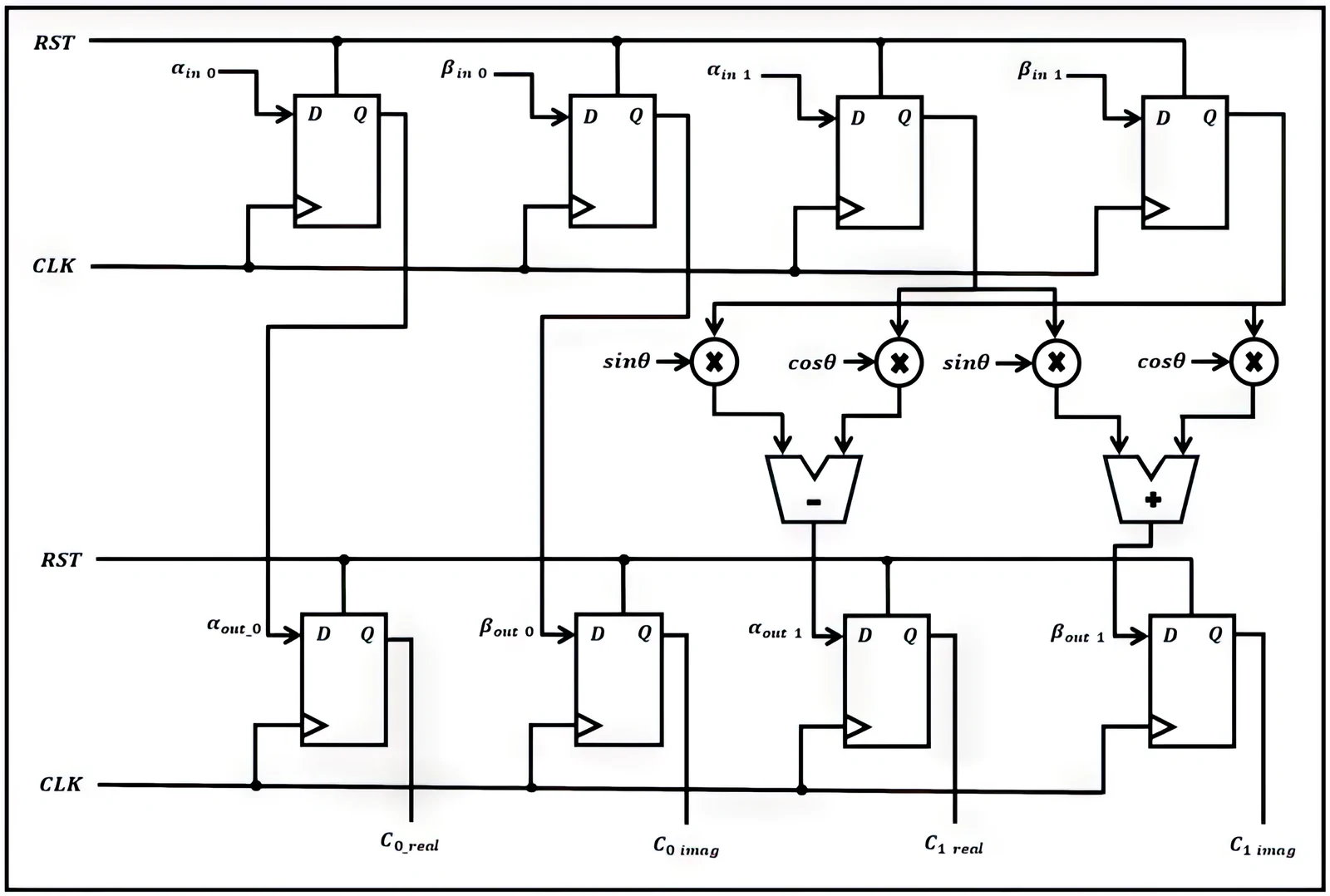
*Phase* Gate emulation micro architecture.

The mathematical representation of the *Phase* gate (P(θ)) transformation is given in Eq 16 and 17. This transformation leaves the |0⟩ state unchanged, and applies a phase (θ) to the |1⟩. The matrix multiplication used to perform a qubit rotation is algebraically simplified as Eq 19 to reduce computational overhead. The micro architecture of the *Phase* gate as per the expression given in Eq 19 is presented in Fig 6. This architecture takes the input probability state and the phase value in radians. The output of this block is the transformed qubit, i.e., $\left|\psi_{out}\right\rangle =C_{\text{out\_0}}|0\rangle +C_{\text{out\_1}}|1\rangle$.

**Fig 7:**
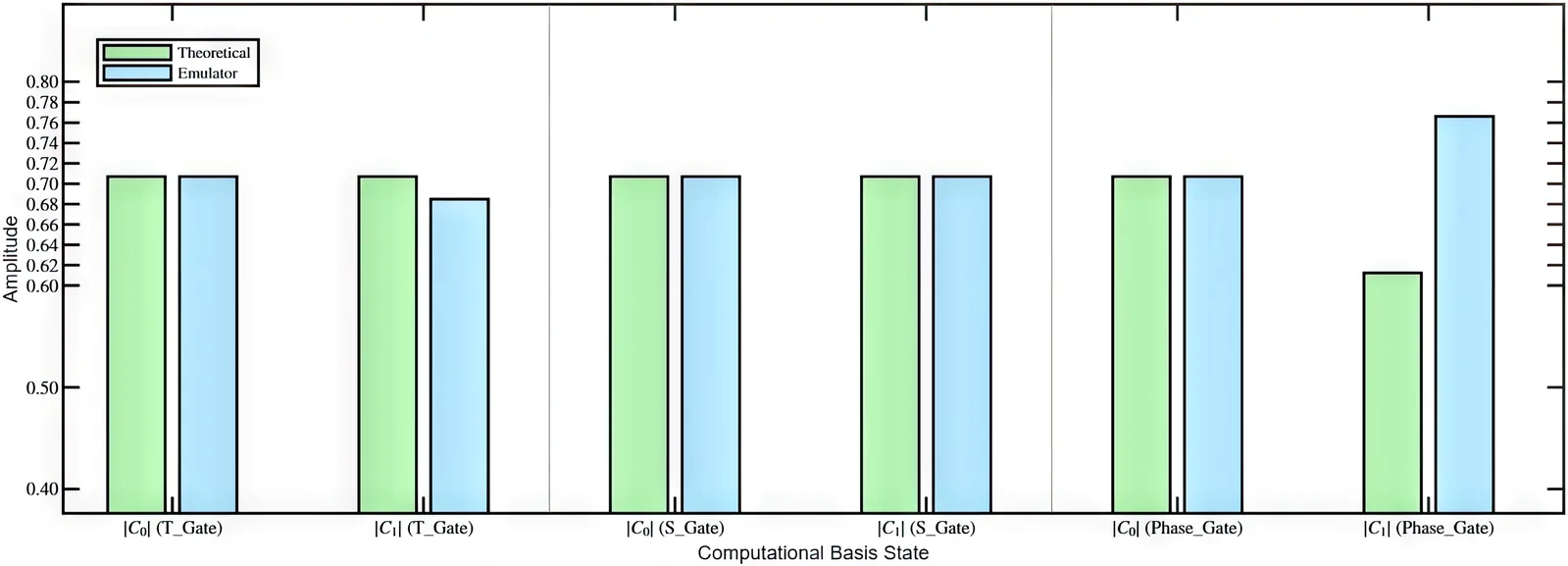
Output state probability distributions for *single-qubit* gates applied to the |+⟩.

For demonstration purposes, the input is initialised as |+⟩ and $\theta =\frac{\pi }{3}$. The trigonometric expressions are represented as signed *Q*(2,6) numbers. The discretization of these expressions due to fixed-point representation affects the accuracy of the emulation output compared to the theoretical results. Figure 7 presents a comparison between the emulation results and the theoretical predictions in terms of quantum state probabilities for the test inputs, i.e., |+⟩ and $\theta =\frac{\pi }{3}$. The Artix-7 based resource utilization summary of *Phase* gate emulation is given in Table 5.


$$\begin{array}{c} |\psi_{out}\rangle =P(\theta )|\psi_{in}\rangle \end{array}$$ (16)

$$\begin{array}{c} \left[\begin{array}{c} C_{\text{out}\_0}\\ C_{\text{out}\_1} \end{array}]=[\begin{array}{cc} 1 & 0\\ 0 & e^{\hat{\iota }\theta } \end{array}][\begin{array}{c} C_{\text{in}\_0}\\ C_{\text{in}\_1} \end{array}]=[\begin{array}{c} C_{\text{in}\_0}\\ e^{\hat{\iota }\theta }C_{\text{in}\_1} \end{array}\right] \end{array}$$ (17)

$$\begin{array}{c} \left[\begin{array}{c} \alpha_{out\_0}+\hat{\iota }\beta_{out\_0}\\ \alpha_{out\_1}+\hat{\iota }\beta_{out\_1} \end{array}]=[\begin{array}{c} \alpha_{in\_0}+\hat{\iota }\beta_{in\_0}\\ e^{\hat{\iota }\theta }(\alpha_{in\_1}+\hat{\iota }\beta_{in\_1}) \end{array}\right] \end{array}$$ (18)

$$\begin{array}{c} ∴e^{\hat{\iota }\theta }=\cos\theta +\hat{\iota }\sin\theta \end{array}$$

$$\begin{array}{c} \left[\begin{array}{c} \alpha_{out\_0}+\hat{\iota }\beta_{out\_0}\\ \alpha_{out\_1}+\hat{\iota }\beta_{out\_1} \end{array}]=[\begin{array}{c} \alpha_{in\_0}+\hat{\iota }\beta_{in\_0}\\ (\alpha_{in\_1}\cos\theta -\beta_{in\_1}\sin\theta )+\hat{\iota }\left(\alpha_{in\_1}\sin\theta +\beta_{in\_1}\cos\theta \right) \end{array}\right] \end{array}$$ (19)

**Table 5:** Resources utilization of the *Phase* gate emulation.

Resource	Utilization(%)	On-Chip Power	Latency
LUTs	323(0.24%)	0.005(3%)W	109 cycles (8 *ns*)
Flip-Flops (FFs)	205(0.08%)		
DSP Blocks	1(0.14%)		
I/O Pins	21(5.25%)		
BRAMs	0(0.00%)		
*F*_max_ (MHz)	129		

*S* and *T* gates are special-purpose *phase* gates that shift the qubits’ azimuthal angle by $\frac{\pi }{2}$ and $\frac{\pi }{4}$ radians, respectively, keeping the probability amplitude intact. Given the applications of these gates in error correction and fault-tolerant quantum computing, separate emulation modules are designed for these operations. The following are the microarchitectures, resource utilization summaries, and theoretical benchmarking of these special-purpose gates.

1. *S-Gate*: The gate rotates the state vector by $\frac{\pi }{2}$ radians about the *z*-axis. The application set of the *S*-Gate includes *phase* correction, Quantum Fourier Transform, and stabilizer circuits.

**Fig 8:**
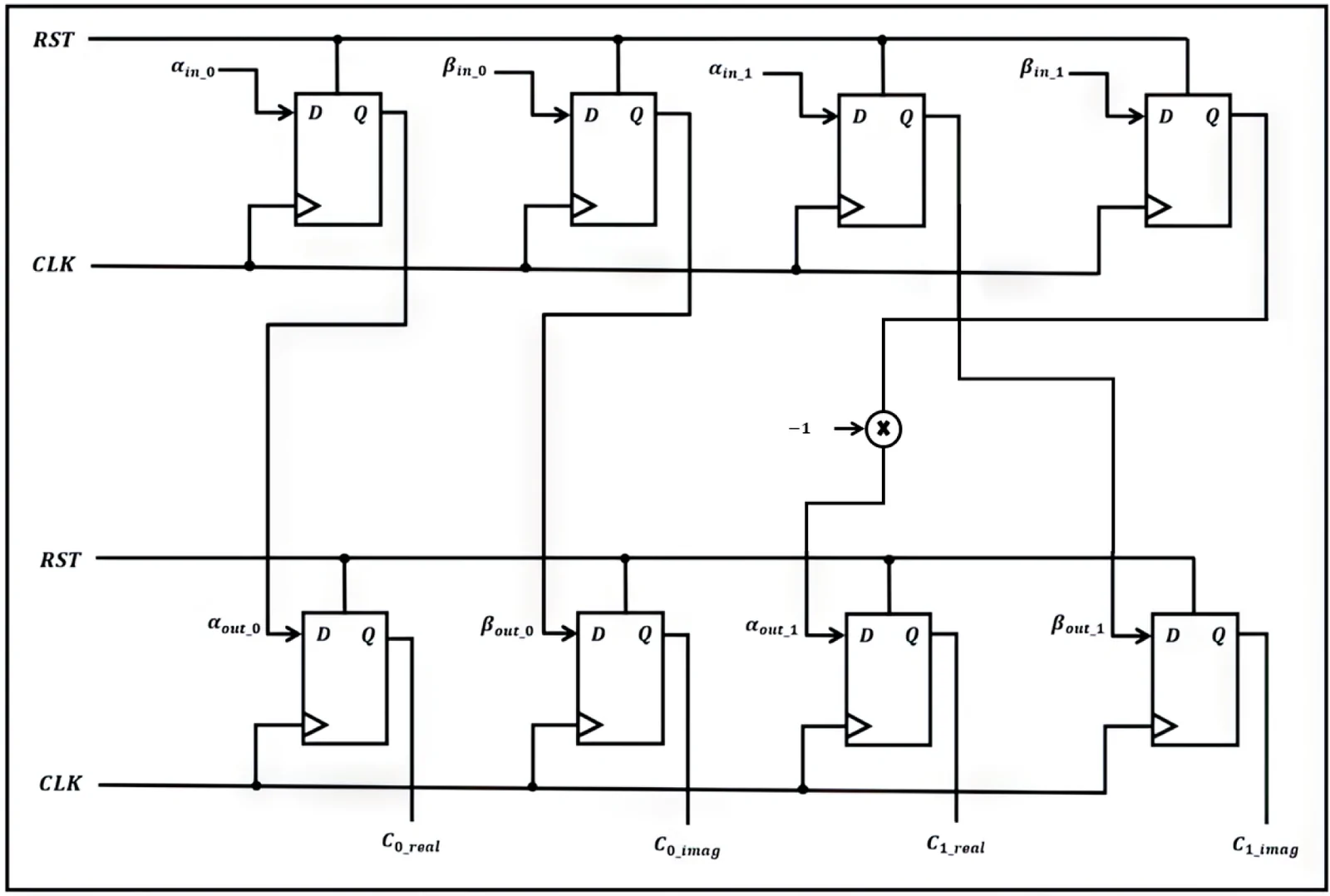
*S*-Gate emulation micro architecture.

The linear operator representation in Hilbert space is given in Eq 20. Whereas, the algebraically simplified form of this unitary gate is presented in Eq 22 and is implemented using the microarchitecture given in Fig 8. The resource utilization summary and output comparison with the theoretical model for input |+⟩ are presented as Table 6 and Fig 7 respectively.


$$\begin{array}{c} |\psi_{out}\rangle =S|\psi_{in}\rangle \end{array}$$ (20)

$$\begin{array}{c} \left[\begin{array}{c} C_{\text{out}\_0}\\ C_{\text{out}\_1} \end{array}]=[\begin{array}{cc} 1 & 0\\ 0 & e^{\hat{\iota }\frac{\pi }{2}} \end{array}][\begin{array}{c} C_{\text{in}\_0}\\ C_{\text{in}\_1} \end{array}]=[\begin{array}{c} C_{\text{in}\_0}\\ e^{\hat{\iota }\frac{\pi }{2}}C_{\text{in}\_1} \end{array}\right] \end{array}$$ (21)

$$\begin{array}{c} \left[\begin{array}{c} \alpha_{out\_0}+\hat{\iota }\beta_{out\_0}\\ \alpha_{out\_1}+\hat{\iota }\beta_{out\_1} \end{array}]=[\begin{array}{c} \alpha_{in\_0}+\hat{\iota }\beta_{in\_0}\\ -\beta_{in\_1}+\hat{\iota }\alpha_{in\_1} \end{array}\right] \end{array}$$ (22)

**Table 6:** Resources utilization of the *S* gate emulation.

Resource	Utilization(%)	On-Chip Power	Latency
LUTs	313(0.23%)	0.004(3%)W	107 cycles(8 *ns*)
Flip-Flops (FFs)	207(0.08%)		
DSP Blocks	1(0.14%)		
I/O Pins	21(5.25%)		
BRAMs	0(0.00%)		
*F*_max_ (MHz)	128		

**Fig 9:**
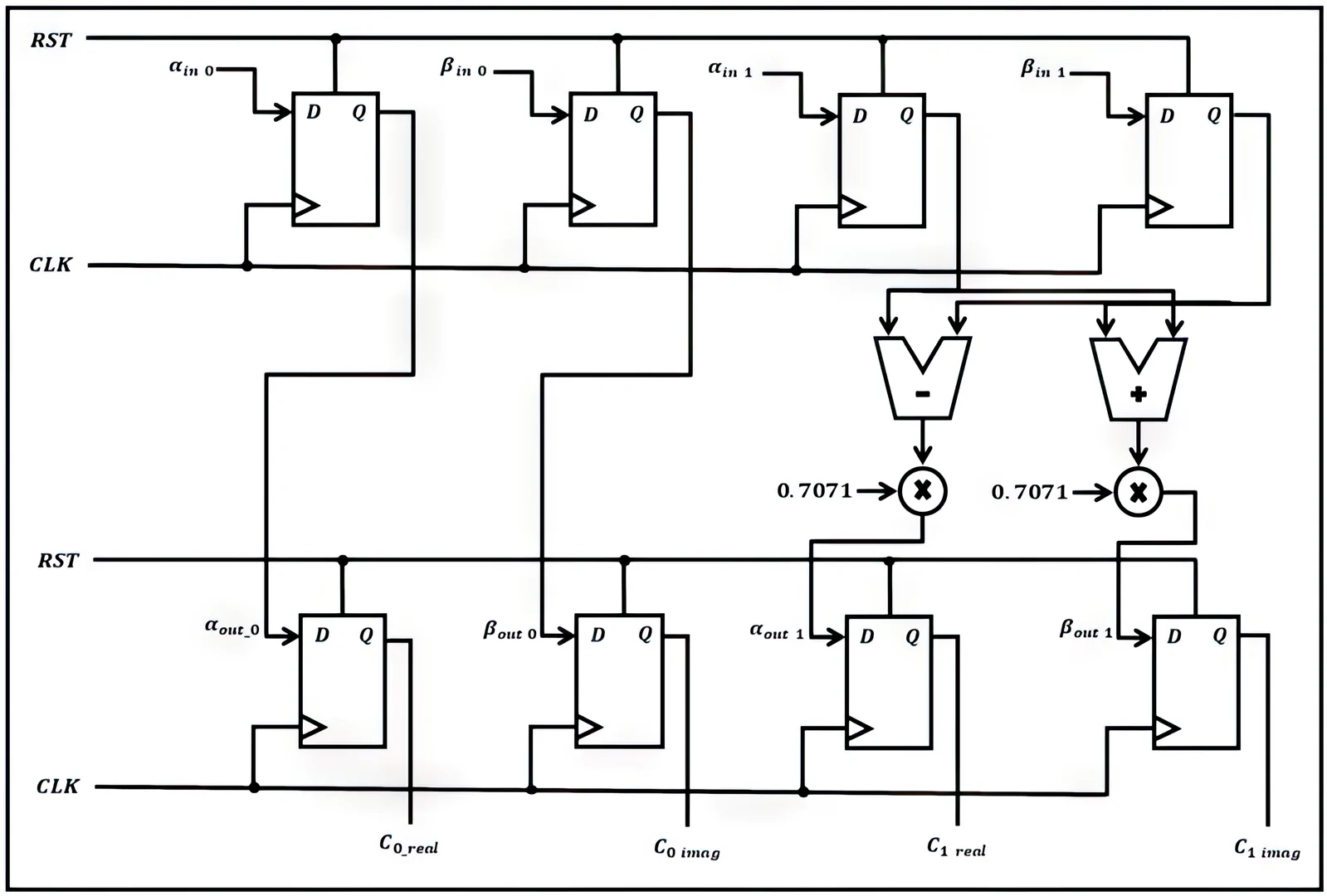
*T*-Gate emulation micro architecture.

2. *T-Gate*: This gate is responsible for the $\frac{\pi }{4}$ phase shift of the input qubit about the *z*-axis. The *T*-Gate is the functional building block of the universal gate set. Eq 23 presents the gate’s unitary matrix. Whereas, the algebraically simplified form of this unitary gate is presented in Eq 25 and is implemented using micro architecture given in Fig 9. The resource utilization summary and output comparison with theoretical model for input |+⟩ is presented as Table 7 and Fig 7 respectively.


$$\begin{array}{c} |\psi_{out}\rangle =T|\psi_{in}\rangle \end{array}$$ (23)

$$\begin{array}{c} \left[\begin{array}{c} C_{\text{out}\_0}\\ C_{\text{out}\_1} \end{array}]=[\begin{array}{cc} 1 & 0\\ 0 & e^{\hat{\iota }\frac{\pi }{4}} \end{array}][\begin{array}{c} C_{\text{in}\_0}\\ C_{\text{in}\_1} \end{array}]=[\begin{array}{c} C_{\text{in}\_0}\\ e^{\hat{\iota }\frac{\pi }{4}}C_{\text{in}\_1} \end{array}\right] \end{array}$$ (24)

$$\begin{array}{c} \left[\begin{array}{c} \alpha_{out\_0}+\hat{\iota }\beta_{out\_0}\\ \alpha_{out\_1}+\hat{\iota }\beta_{out\_1} \end{array}]=[\begin{array}{c} \alpha_{in\_0}+\hat{\iota }\beta_{in\_0}\\ \frac{1}{\sqrt{2}}\left(\alpha_{in\_1}-\beta_{in\_1}\right)+\frac{\hat{\iota }}{\sqrt{2}}\left(\alpha_{in\_1}+\beta_{in\_1}\right) \end{array}\right] \end{array}$$ (25)

**Table 7:** Resources utilization of the *T* gate emulation.

Resource	Utilization(%)	On-Chip Power	Latency
LUTs	310(0.23%)	0.006(4%)W	109 cycles(8 *ns*)
Flip-Flops (FFs)	191(0.07%)		
DSP Blocks	1(0.14%)		
I/O Pins	21(5.25%)		
BRAMs	0(0.00%)		
*F*_max_ (MHz)	129		

The emulation architecture of the *Phase*, *S*, and *T* gates represents the algebraic simplification of complex matrix multiplications involved in applying unitary operators.

#### 3.3.2 Two qubit gate emulation.

The CNOT gate is a fundamental 2-qubit operation that enables entanglement generation, completes the universal gate set, and acts as an analogue to the classical *Xor* gate in the quantum domain. In the CNOT gate, the control qubit governs the transition of the target qubit. Mathematically, it is represented as a 4×4 matrix multiplied by the tensor product of input qubits, i.e., control and target qubits, as shown in Eq 27. A separate module for the tensor product is designed to serve as a prerequisite for the CNOT gate. The architecture is based on the algebraic simplification in complex arithmetic as defined in Eq 3 and 4.


$$\begin{array}{c} \left|\psi_{\text{out}}\rangle ={𝑈}_{\text{CNOT}}\}(|\psi_{\text{control}}\rangle ⊗|\psi_{\text{target}}\rangle \right) \end{array}$$ (26)

$$\begin{array}{c} \left[\begin{array}{c} C_{{\text{out}}_{\_}00}\\ C_{{\text{out}}_{\_}01}\\ C_{{\text{out}}_{\_}10}\\ C_{{\text{out}}_{\_}11} \end{array}]=[\begin{array}{cccc} 1 & 0 & 0 & 0\\ 0 & 1 & 0 & 0\\ 0 & 0 & 0 & 1\\ 0 & 0 & 1 & 0 \end{array}][\begin{array}{c} C_{{\text{in}}_{\_}00}\\ C_{{\text{in}}_{\_}01}\\ C_{{\text{in}}_{\_}10}\\ C_{{\text{in}}_{\_}11} \end{array}]=[\begin{array}{c} C_{{\text{in}}_{\_}00}\\ C_{{\text{in}}_{\_}01}\\ C_{{\text{in}}_{\_}11}\\ C_{{\text{in}}_{\_}10} \end{array}\right] \end{array}$$ (27)

$$\begin{array}{c} \left[\begin{array}{c} \alpha_{out\_00}+\hat{\iota }\beta_{out\_00}\\ \alpha_{out\_01}+\hat{\iota }\beta_{out\_01}\\ \alpha_{out\_10}+\hat{\iota }\beta_{out\_10}\\ \alpha_{out\_11}+\hat{\iota }\beta_{out\_11} \end{array}]=[\begin{array}{c} \alpha_{in\_00}+\hat{\iota }\beta_{in\_00}\\ \alpha_{in\_01}+\hat{\iota }\beta_{in\_01}\\ \alpha_{in\_11}+\hat{\iota }\beta_{in\_11}\\ \alpha_{in\_10}+\hat{\iota }\beta_{in\_10} \end{array}\right] \end{array}$$ (28)

**Fig 10:**
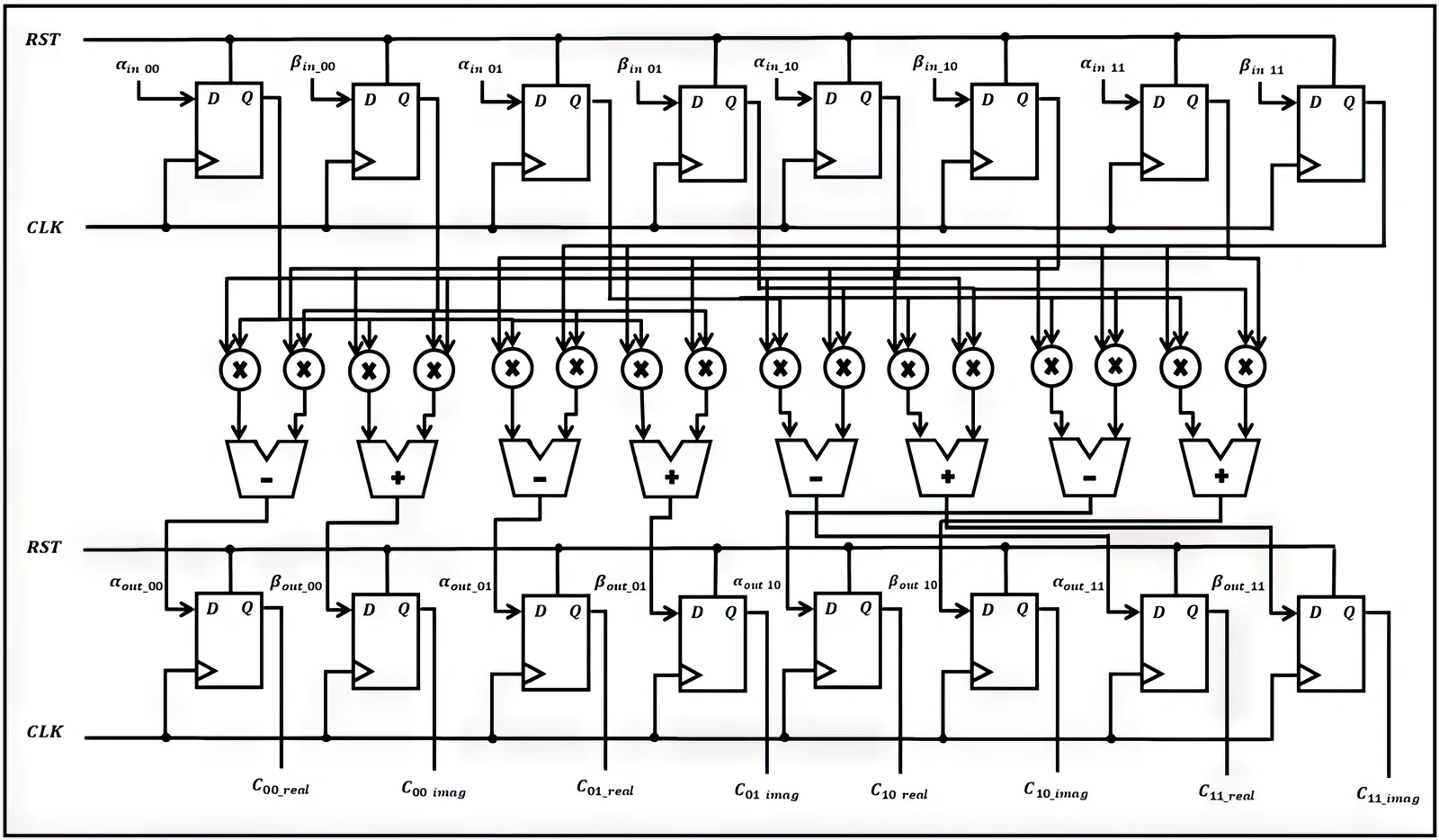
*CNOT-Gate* emulation micro architecture.

**Fig 11:**
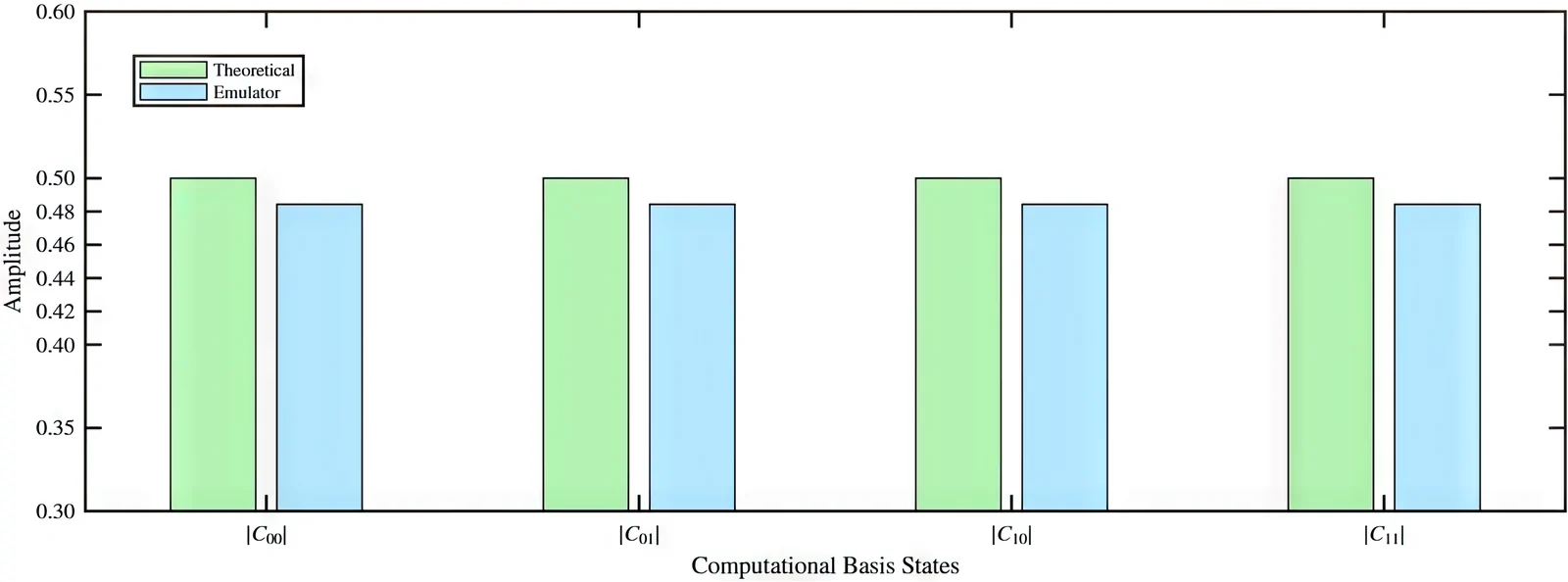
Output state probability distributions for the CNOT gate.

To ensure accuracy and resource efficiency, the behavioural architecture of the gate is emulated as shown in Fig 10, and the corresponding resource utilization metrics are summarized in Table 8. The results of the gate with inputs |+⟩⊗|−⟩ are elaborated in Fig 11.

**Table 8:** Resources utilization of the *CNOT* gate emulation.

Resource	Utilization(%)	On-Chip Power	Latency
LUTs	1921(1.43%)	0.024(15%)W	136 cycles(8 *ns*)
Flip-Flops (FFs)	748(0.28%)		
DSP Blocks	4(0.54%)		
I/O Pins	70(17.50%)		
BRAMs	0(0.00%)		
*F*_max_ (MHz)	140		

A common implementation insight in all the above proposed architectures is that the Block RAM (BRAM) was not employed. The reason for this design choice is that the small state space of the targeted quantum systems favors register-based storage, enabling single-cycle access and efficient amplitude permutation without incurring BRAM access overhead.

### 3.4 Finite state machine

The FSM is a control module that invokes the memory, ALU, and measurement blocks based on the user-defined quantum circuit. In line with the sequential computational model of the quantum circuit, for a given quantum system, the FSM serially instantiates the quantum gates for input transformations, stores the resultant state in quantum memory or directs the measurement block to produce the classical output.

**Fig 12:**
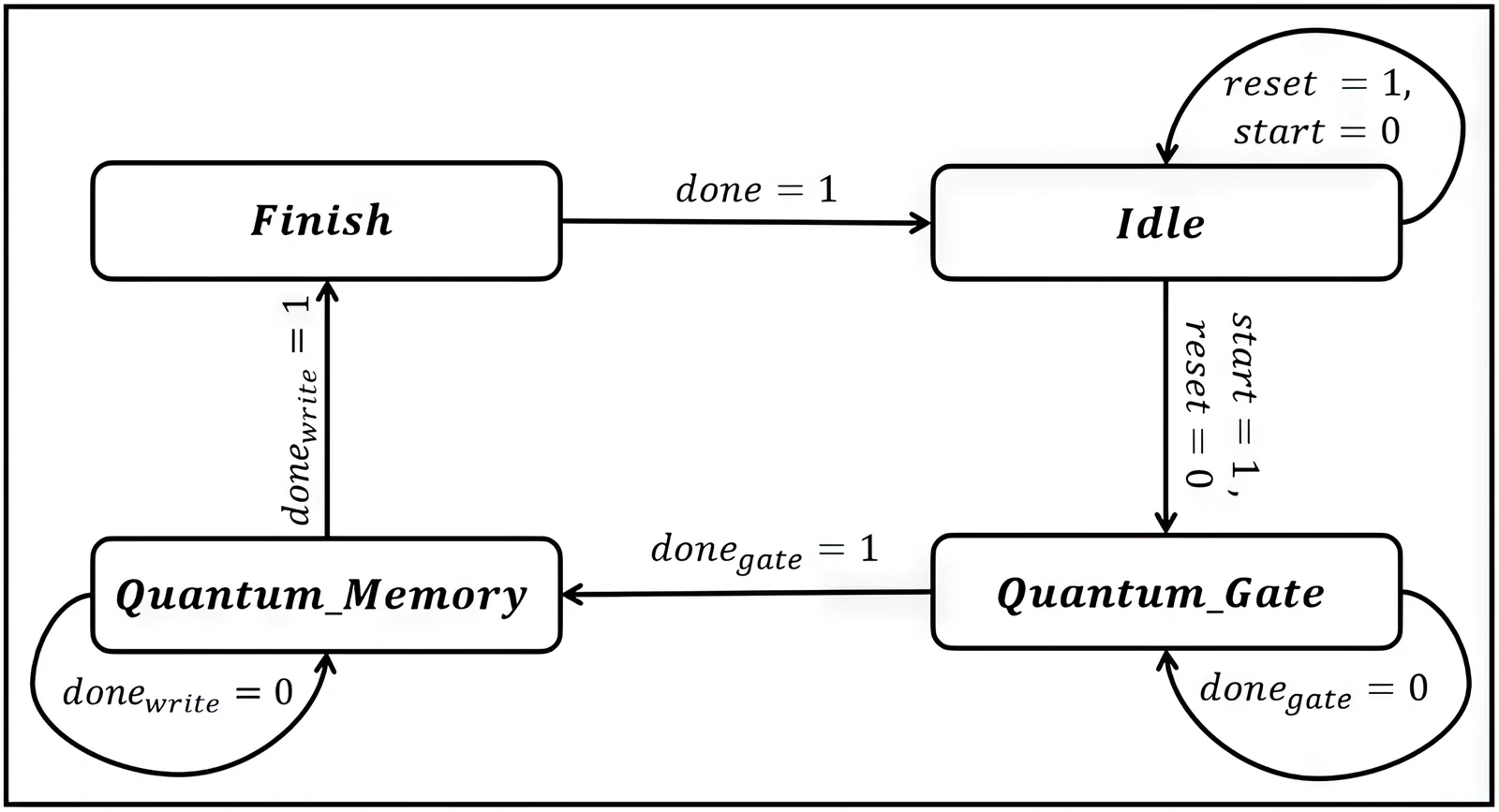
FSM for quantum gate emulation.

Figure 12 presents a generalised FSM for a single gate emulation. The FSM starts on the positive edge labeled *Start*. The input qubits are transitioned according to the selected gate (*Phase*, *S*, *T*, CNOT). The output is then stored in the register file, and the process concludes at the *Finish* stage.

Figure 3 defines an independent setup capable of executing quantum circuits since it can emulate the universal gate set. To validate the claim, a computational procedure for the emulation of an arbitrary unitary single gate is presented in the subsequent section.

### 3.5 Arbitrary single-qubit gate emulation

The universality in single-qubit transformation requires two *single-qubit* gates (rotation) as defined in Eq 29.


$$\begin{array}{c} G=\{R_{l}(\beta ),\;R_{m}(\alpha )\} \end{array}$$ (29)

where *l* and *m* are non-parallel axes of the Bloch sphere and α,β∈[0,2π) [13]. In classical mechanics, the Euler Rotation Theorem states that any orientation of a 3D unit vector on a sphere can be achieved by three rotations, i.e., (θ,ϕ,λ), about two orthogonal (non-parallel) axes. Relating the Euler rotation theorem to the condition for a universal set, any arbitrary state transformation of a qubit on the Bloch sphere can be accomplished by rotations about two non-parallel axes, i.e., rotation along the *z* and *x* axes.

Therefore, by virtue of the Euler rotation theorem, the expression of single qubit transformation universality is given in Eq 30.


$$\begin{array}{c} U(\theta ,\phi ,\lambda )=R_{z}(\phi )\;R_{x}(\theta )\;R_{z}(\lambda ) \end{array}$$ (30)

The above expression can then be simplified as standard quantum operations, i.e., Phase and Hadamard gates, as given in Eq 31.


$$\begin{array}{c} U=P(\phi )\;H\;P(\theta )\;H\;P(\lambda ) \end{array}$$ (31)

Where P(ϕ) and P(λ) refer to rotation along the z-axis, and HP(θ)H is rotation around the x-axis. This *Z*-*X*-*Z* decomposition is used in industry-standard frameworks like Qiskit, and is adopted in the proposed emulator. For the emulation of a *single-qubit* unitary gate, i.e., U(θ,ϕ,λ), *Phase* and *Hadamard* gates will be instantiated in the following order.

1. P(λ): Adjusts the relative phase of the input state.
2. HP(θ)H: Changes the latitude of the qubit
3. P(ϕ): Adjusts the longitude of the qubit

**Fig 13:**
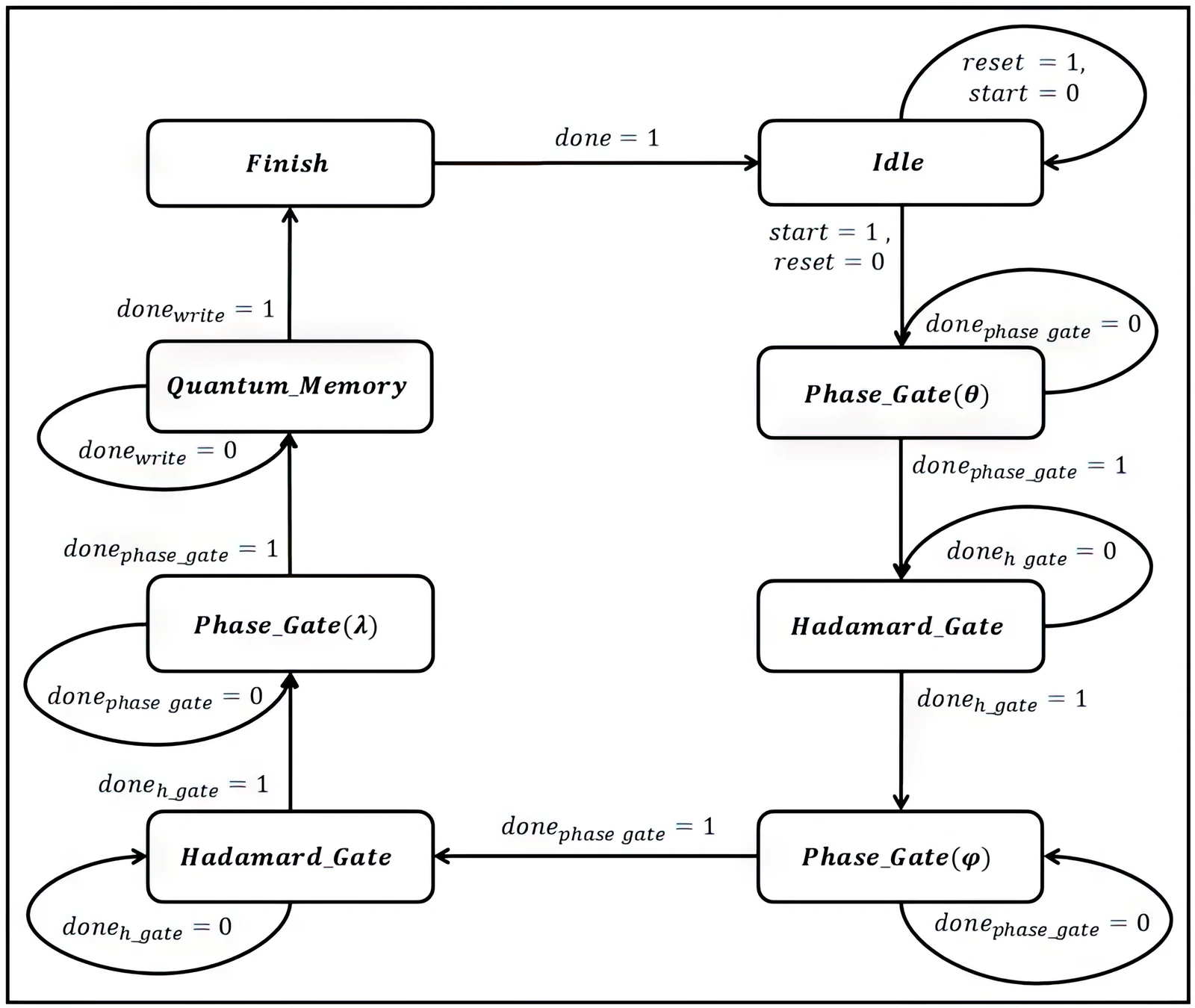
Block diagram of the universal quantum circuit.

**Fig 14:**
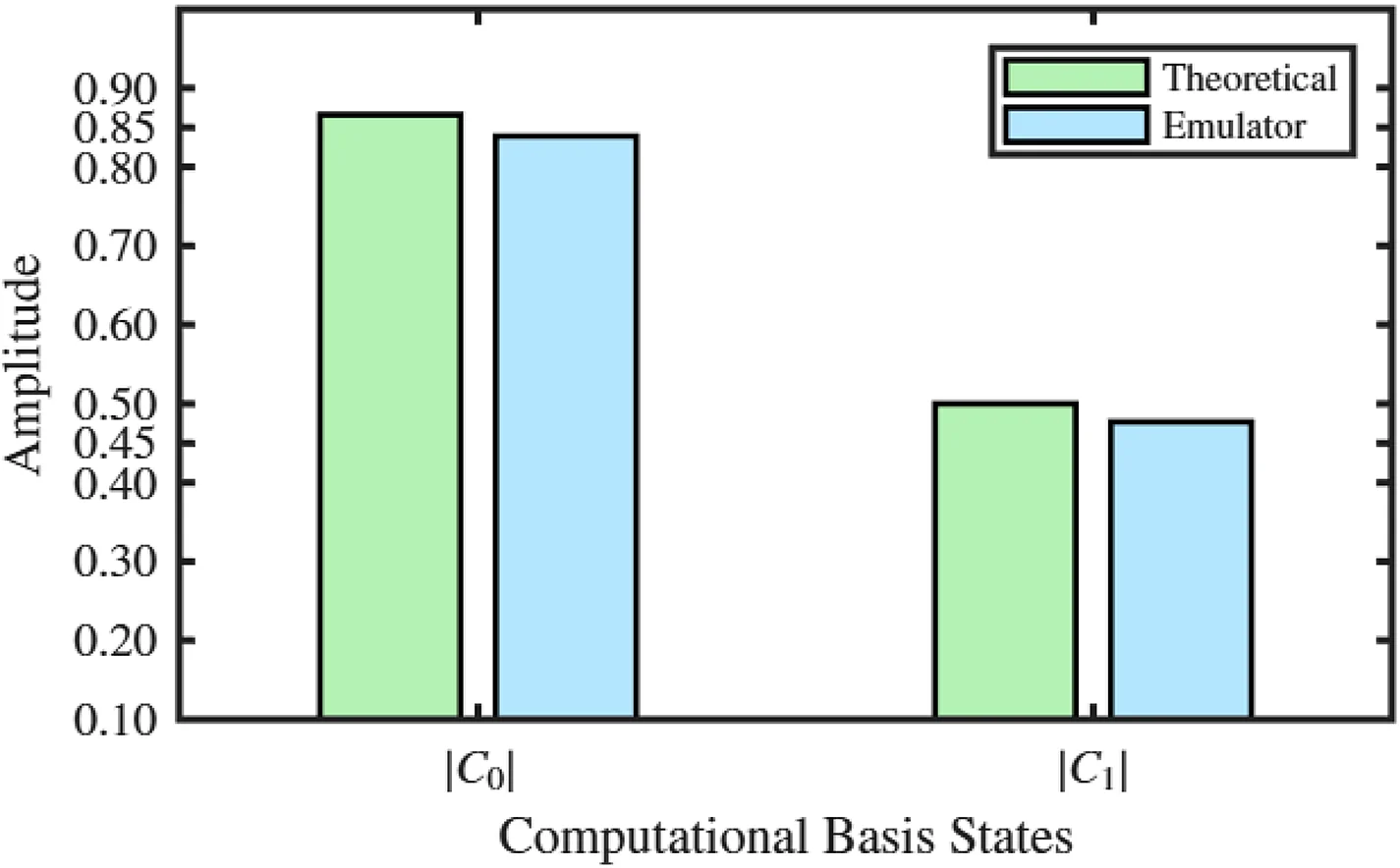
Output states for arbitrary single-qubit transformation emulation.

Figure 13 presents the FSM for the arbitrary single qubit transformation. For experimental verification Fig 14 the results of applying a single-qubit arbitrary transformation to the input state |0⟩, using the universal parameter set (θ,ϕ,λ) with angles π/3, π/2, and π/4 respectively.

The subsequent section presents the validation of the proposed architecture by implementing rudimentary quantum circuits.

## 4 Implementation of quantum algorithms

To verify the correctness of the proposed emulation framework, five foundational circuits from quantum computing are executed, i.e., Bell state preparation, Deutsch’s algorithm, Superdense coding, Grover’s search, and BB84 Quantum Key Distribution (QKD). The premise behind choosing these use cases is to confirm superposition handling, represent entanglement within the computational architecture, and assess functional accuracy. The implementation details of these quantum circuits are as follows:

### 4.1 Bell states generation

**Fig 15:**
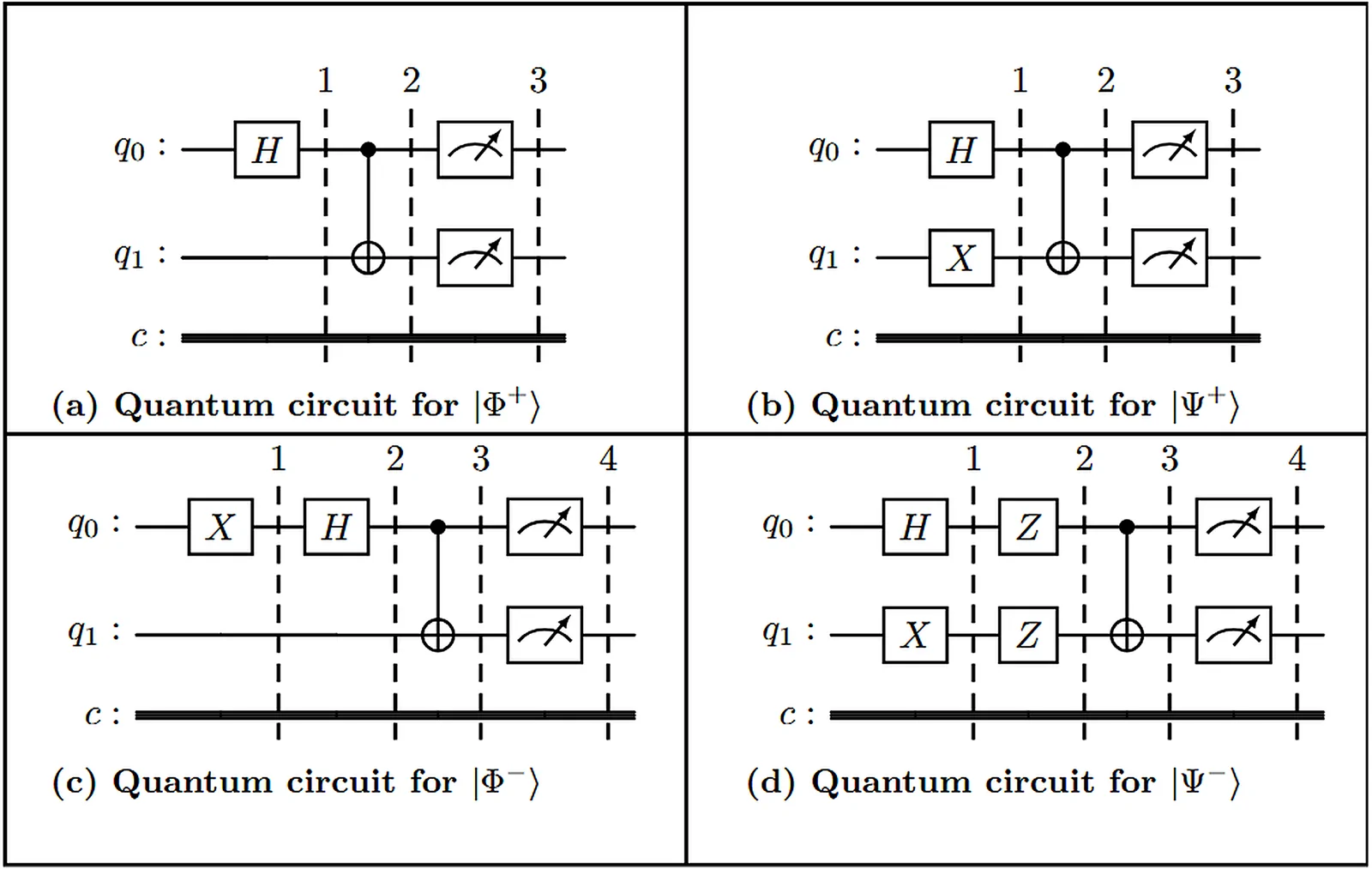
Quantum circuits for *Bell-States.*

Bell state generation is a 2-qubit circuit that first creates a superposition by applying a *Hadamard* gate on the first qubit, followed by a CNOT gate to entangle it with the second qubit, producing states as shown in Fig 15. The resulting entangled pairs serve as fundamental resources for protocols such as quantum teleportation and superdense coding. The successful emulation of Bell circuits verifies the capability of the proposed architecture to realize entanglement, one of the essential requirements for a universal gate set.

**Fig 16:**
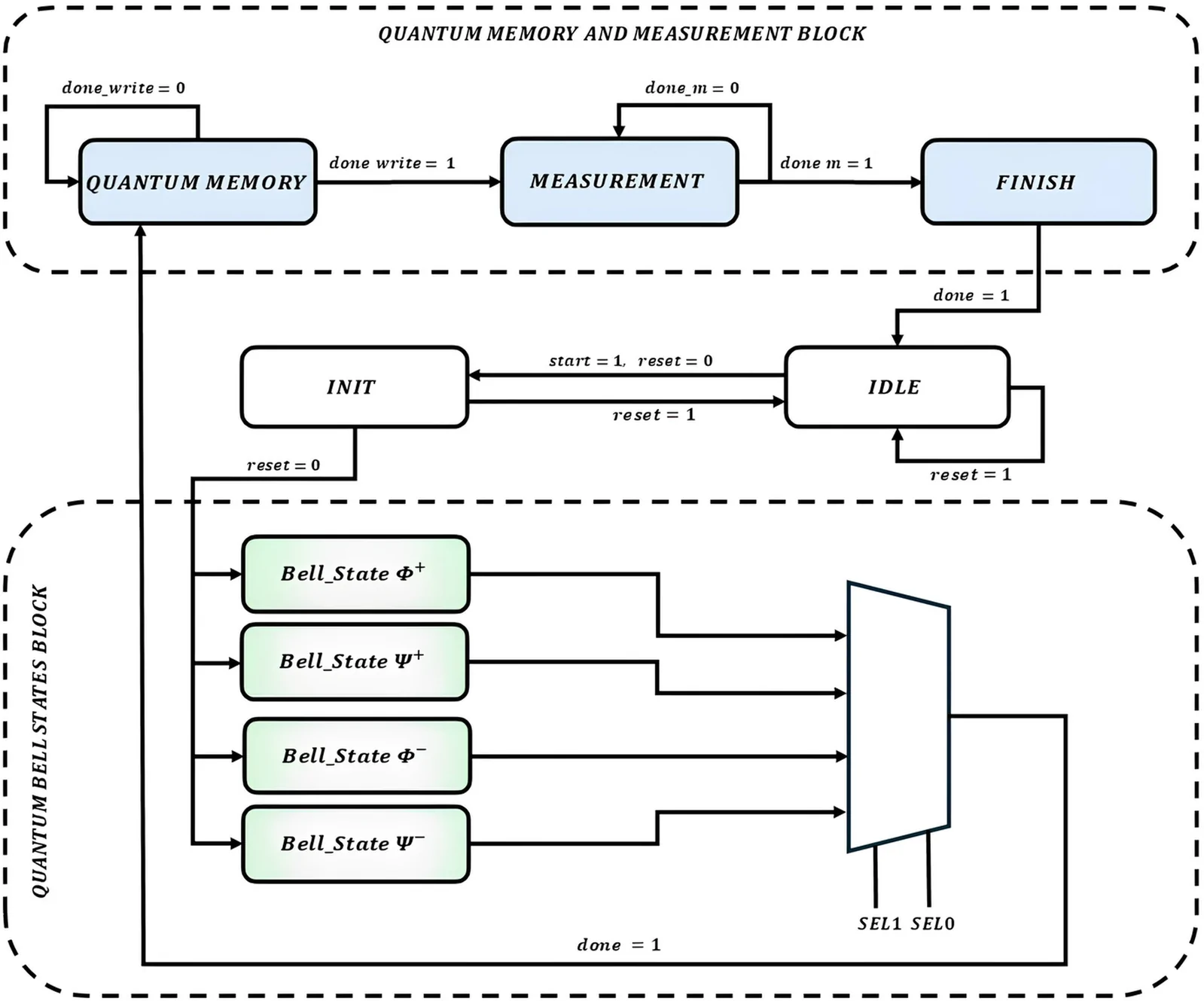
Control-driven execution flow of emulator modules during Bell-state preparation.

**Fig 17:**
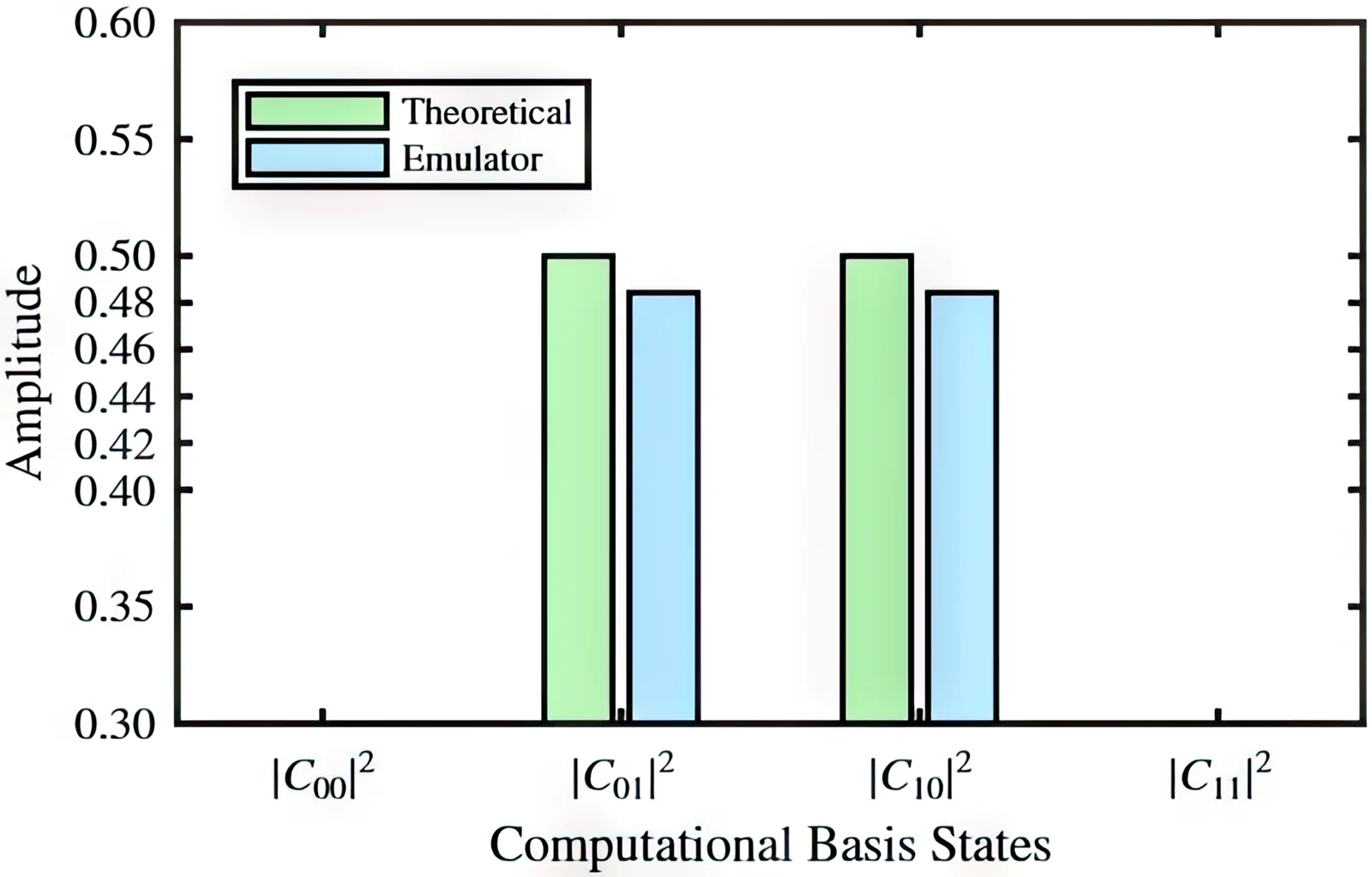
Amplitude comparison of ${|\Phi }^{+}\rangle$.

**Fig 18:**
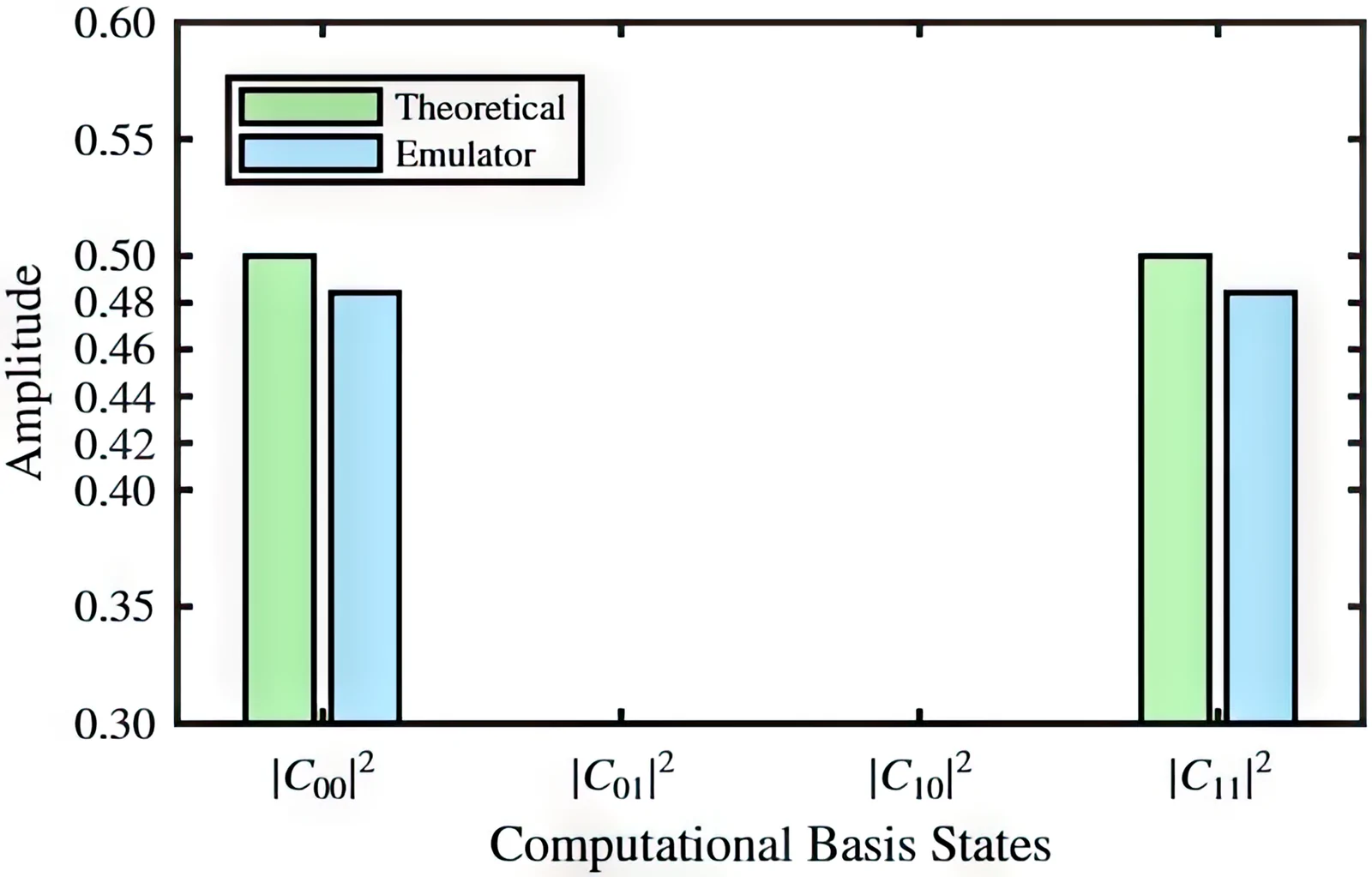
Amplitude comparison of ${|\Psi }^{+}\rangle$.

**Fig 19:**
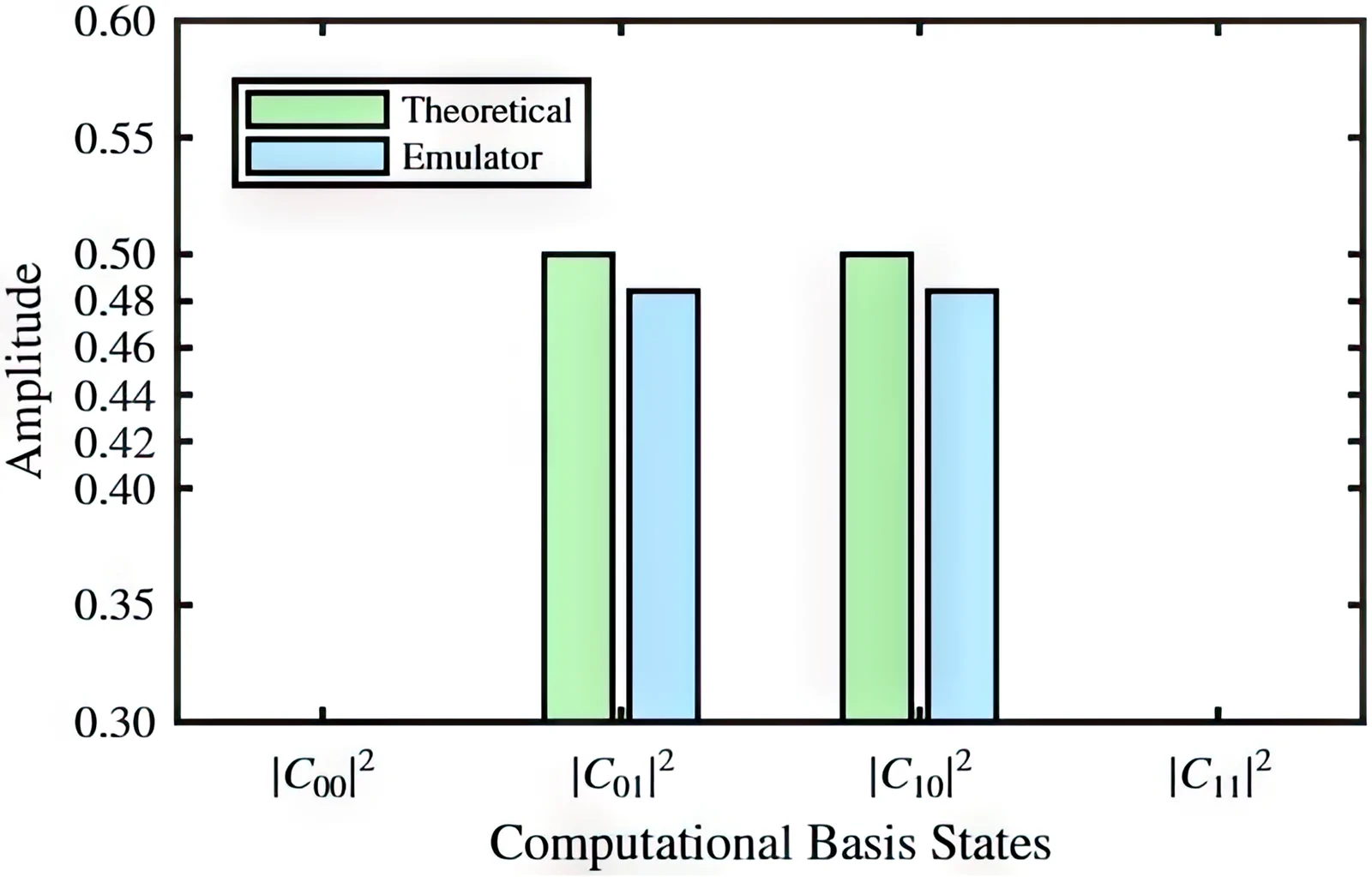
Amplitude comparison of ${|\Psi }^{-}\rangle$.

**Fig 20:**
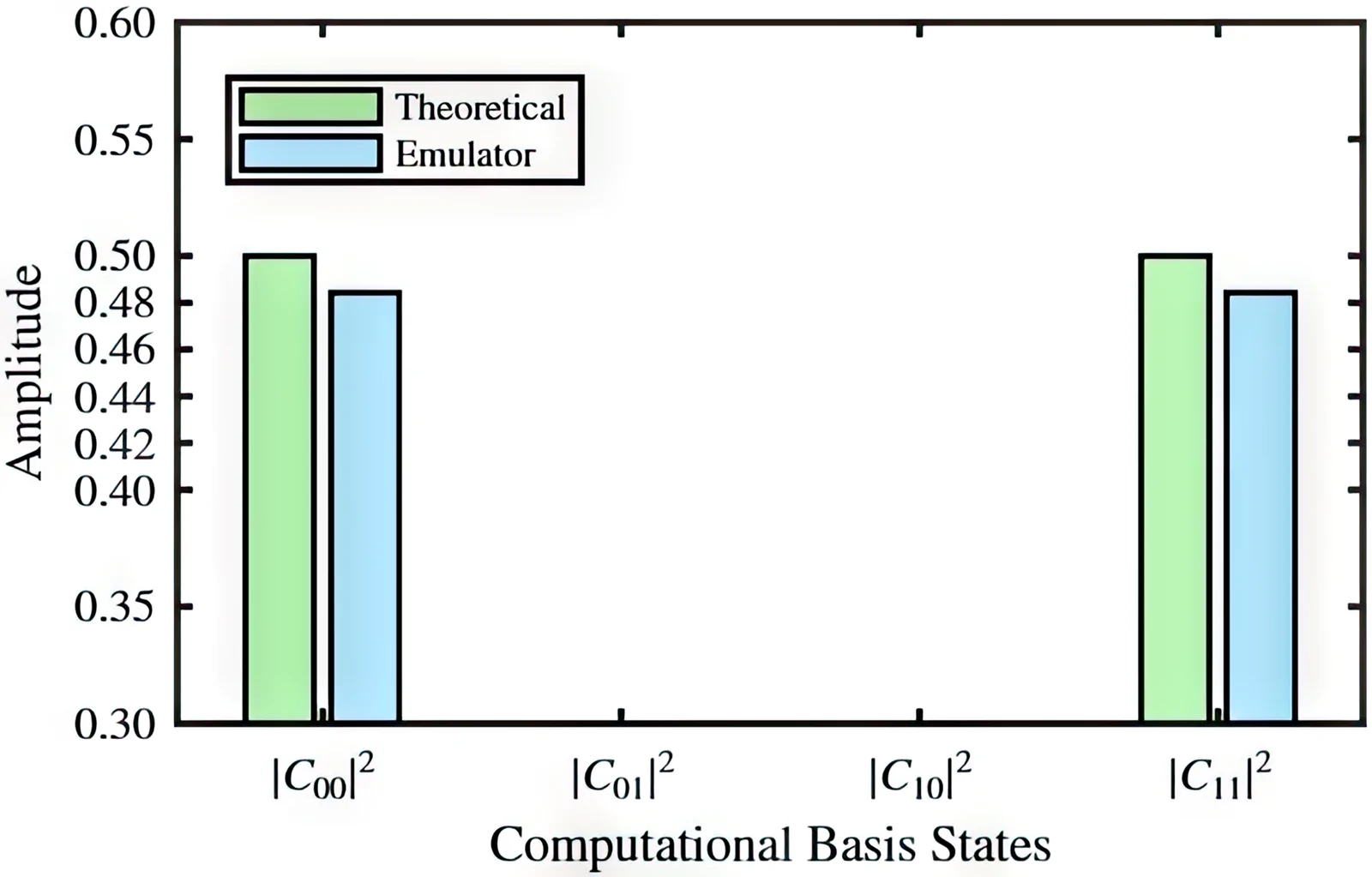
Amplitude comparison of ${|\Phi }^{-}\rangle$.

Figure 16 depicts the control-driven execution flow diagram that incorporates four Bell states ($|\Phi^{+}\rangle$, $|\Psi^{+}\rangle$, $|\Phi^{-}\rangle$, and $|\Psi^{-}\rangle$). The output of any selected Bell state is first stored in the quantum memory and subsequently directed to the measurement module for classical data generation. The coordinated control logic guarantees precise timing and accurate emulation throughout all operational stages. The emulation results for the Bell states closely align with theoretical benchmarking, as shown in Figs 17–20.

### 4.2 Deutsch algorithm

Deutsch’s algorithm is a two-qubit rudimentary circuit that determines whether a Boolean function *y* = *f*(*x*) is constant or balanced in a time-efficient manner. The algorithm flow includes the translation of the classical function (*f*(*x*)) into an oracle that is then embedded in Deutsch’s circuit.

**Fig 21:**
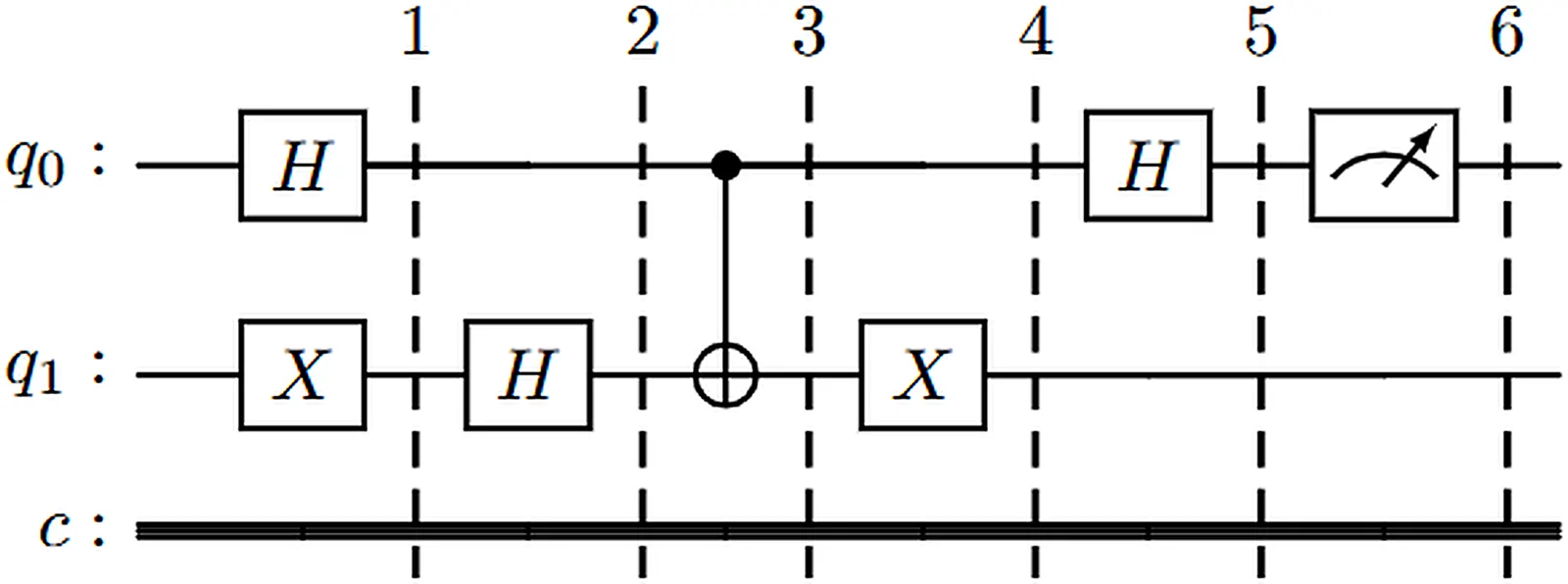
Quantum circuit for *Deutsch’s* algorithm.

**Fig 22:**
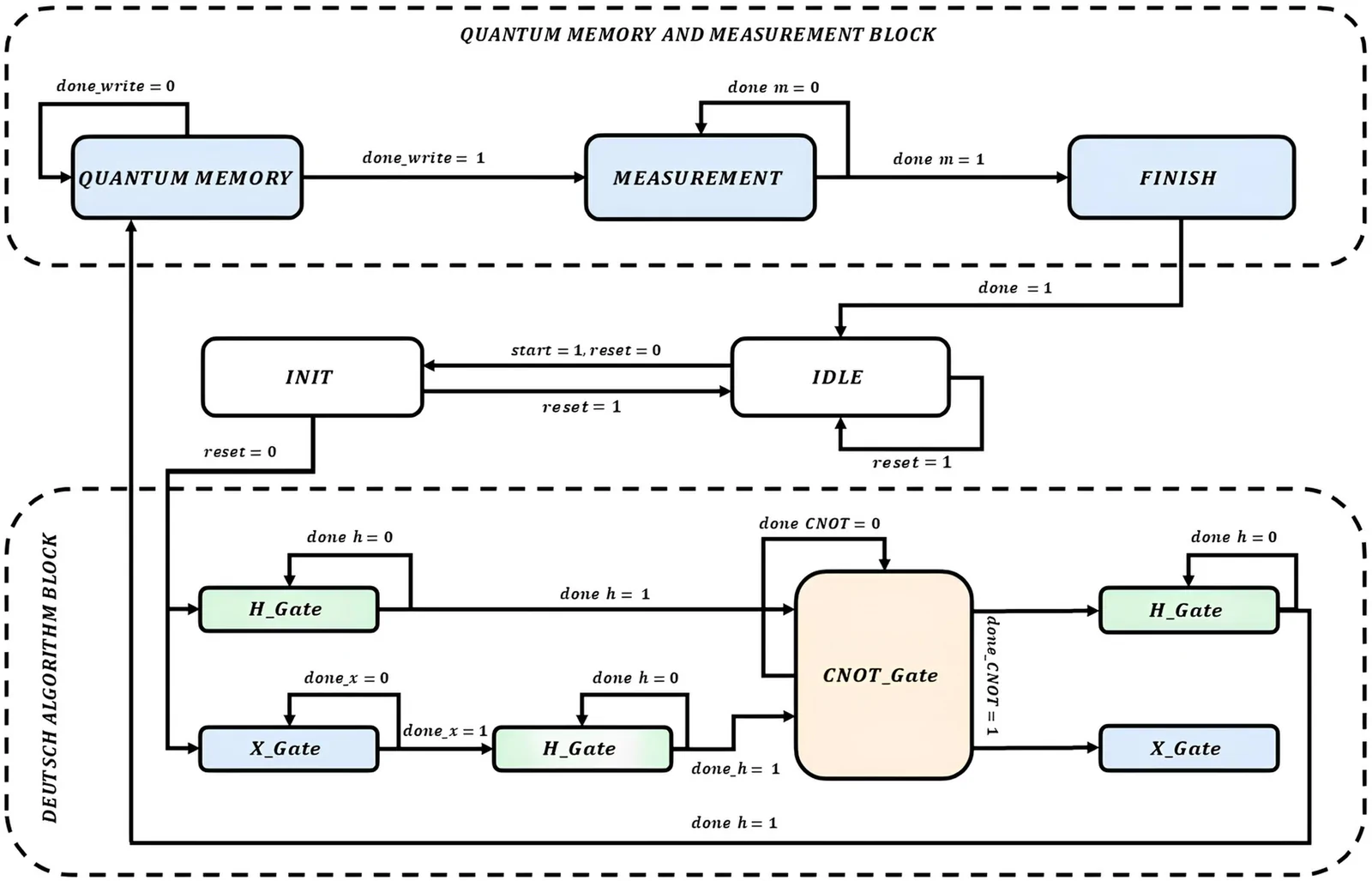
Control-driven execution flow of emulator modules for the Deutsch’s algorithm.

**Fig 23:**
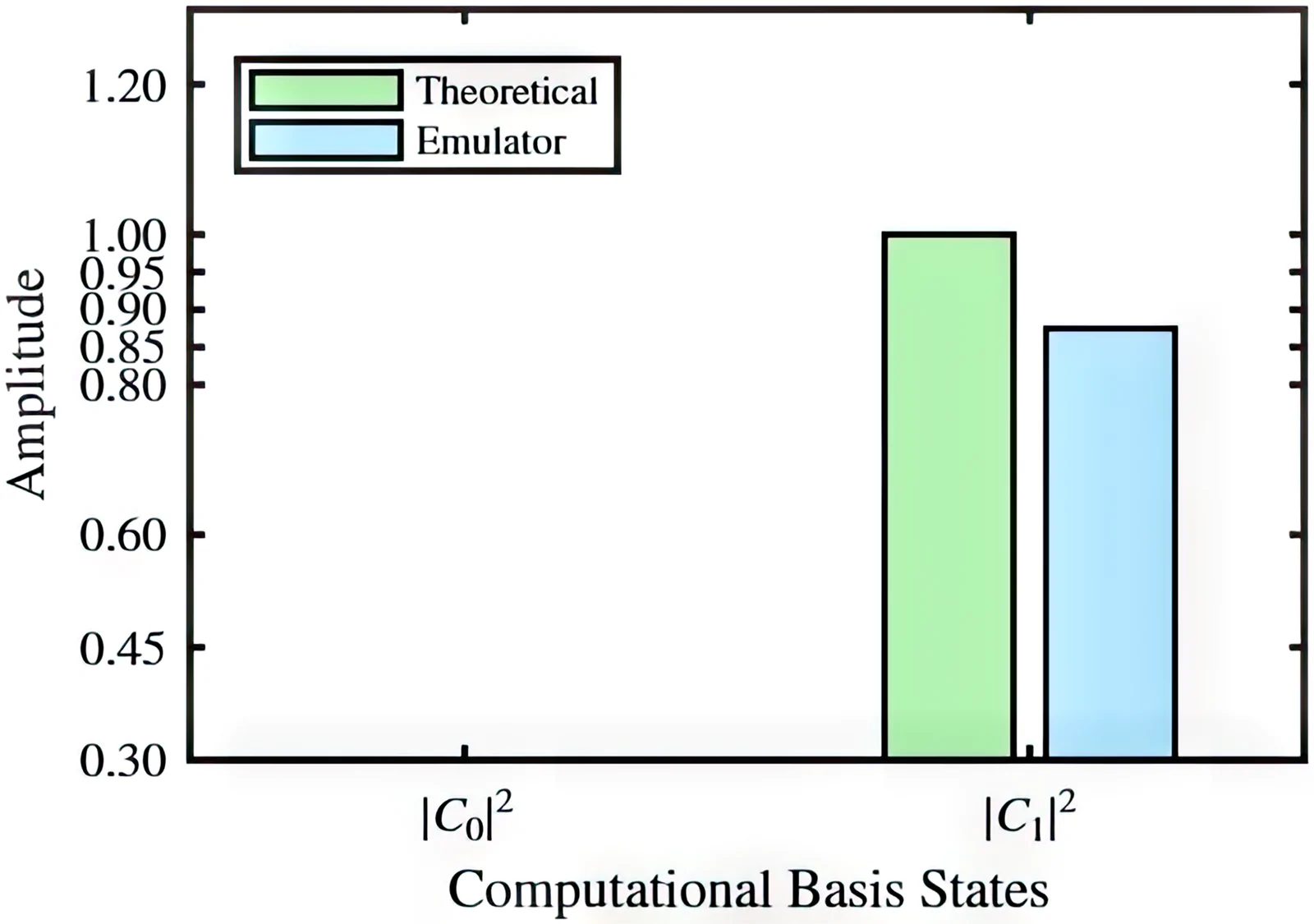
Amplitude comparison for Deutsch algorithm (*Balance* function).

For the validation purposes, the phase oracle of the *Xnor* gate is tested on the Deutsch algorithm as presented in a quantum circuit and control-driven execution flow diagram as given in Fig 21 and 22 respectively. Since *Xnor* is a balanced function, the expected measurement result of the least significant qubit should be 1. The results for the emulation circuit in comparison with the theoretical results are presented in Fig 23.

### 4.3 Superdense coding circuit

Superdense coding enables communication of two classical bits, i.e., (*c*,*d*), through a single-qubit over a quantum channel between Alice (Sender) and Bob (Receiver) at the expense of one entangled bit, i.e., $|\Phi^{+}\rangle$. By applying conditional Pauli operations $X^{c}Z^{d}$ to her half of the entangled pair, Alice encodes four distinct orthogonal states, which Bob subsequently decodes through a Bell-basis measurement (CNOT followed by a *Hadamard* gate).

**Fig 24:**
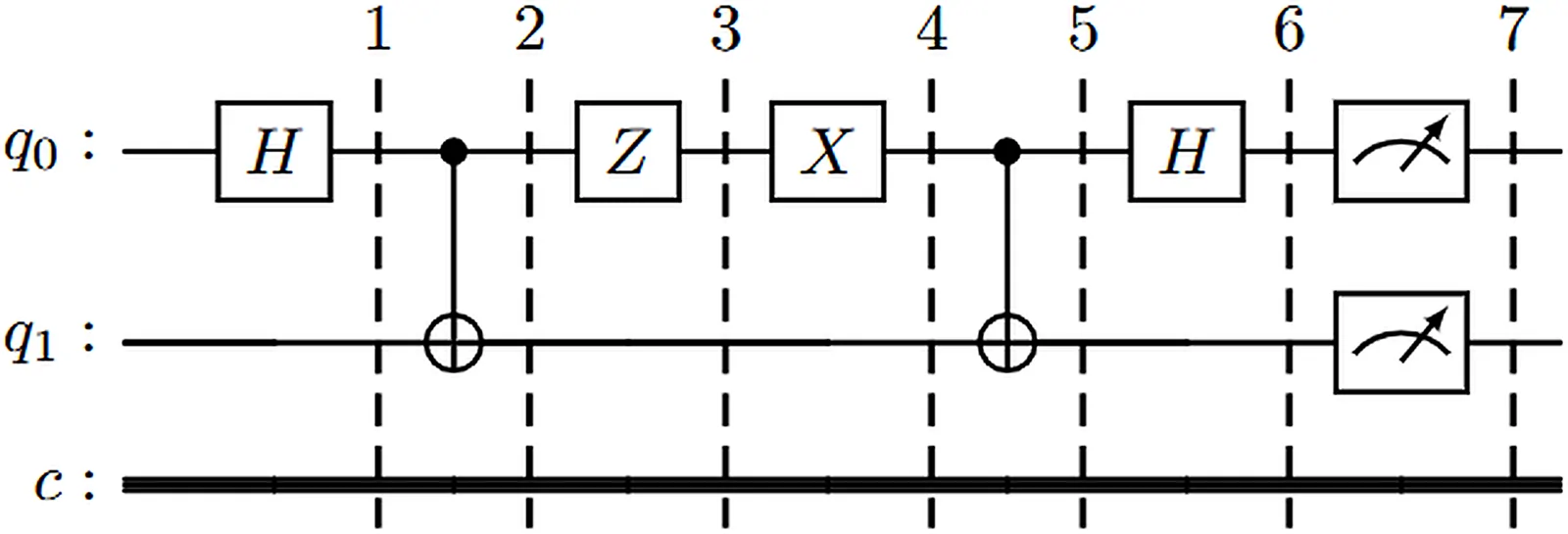
Quantum circuit for Superdense coding.

Figure 24 illustrates the circuit for communication of (*c* = 1,*d* = 1) from the sender to the receiver. In the given circuit, the Bell state $|\Phi^{+}\rangle$ is prepared at barrier 2, followed by the application of $X^{1}Z^{1}$. Barrier 4 depicts the communication channel, and Bell state measurement is performed at the receiver end on barrier 6.

**Fig 25:**
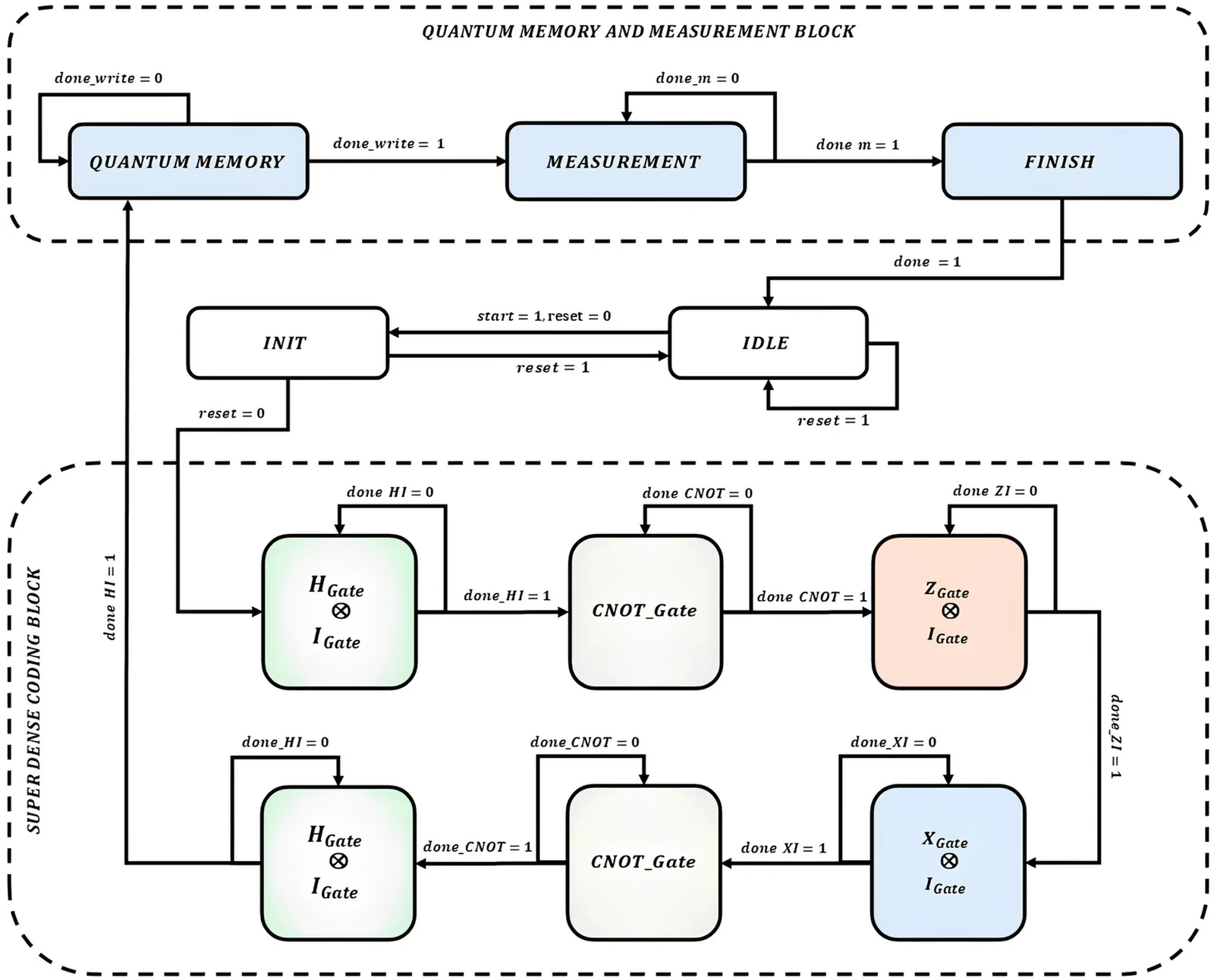
Control-driven execution flow of emulator modules for the Superdense coding circuit.

Since an emulated quantum communication channel is beyond the scope of the proposed work, both the sender’s and receiver’s sides are defined in a unified FSM, where conditional Pauli operations on *q*_0_ result in classical information (*c*,*d*) translation onto the measurement results of qubits *q*_1_ and *q*_0_ respectively. The controller-driven execution flow of superdense coding for transmitting (*c*,*d*)=(1,1) with both qubits initialized to |0⟩ is shown in Fig 25.

**Fig 26:**
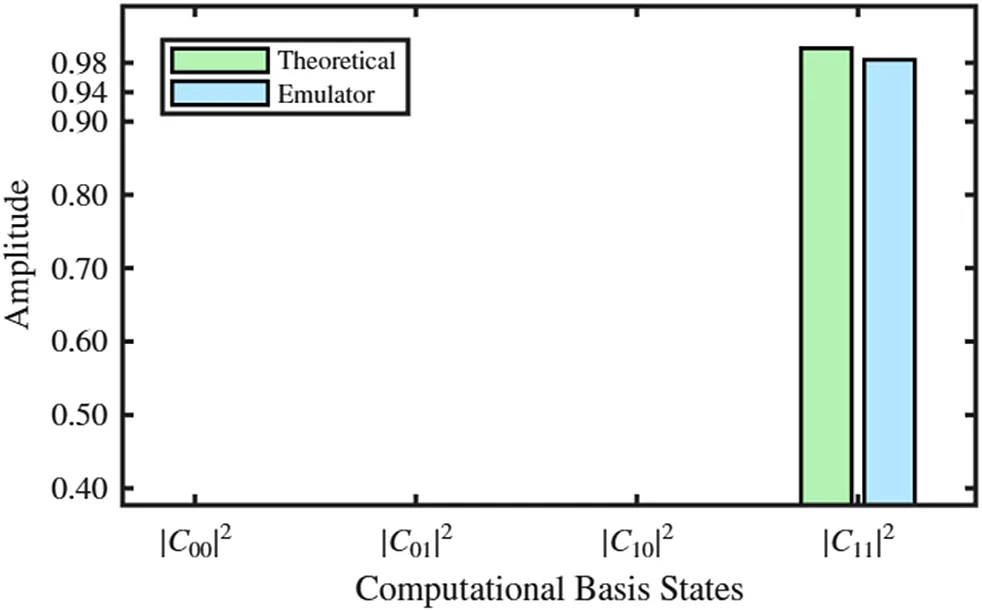
Amplitude comparison of Superdense coding circuit with *c* = 1 and *d* = 1.

According to the circuit analysis, the measurement results should predominantly give the classical output of 11. Fig 26 presents the output of emulated superdense coding that is aligned with the theoretical outcome.

The proposed two-qubit emulation module is designed as a reusable building block that can be instantiated multiple times to support the parallel execution of independent quantum circuits. This scalability is demonstrated in section 5 through the parallel implementation of eight independent two-qubit Superdense Coding circuits for the communication of a 16 -bit binary word, rather than through the emulation of a single 16 -qubit quantum state.

### 4.4 Grover’s search algorithm

**Fig 27:**
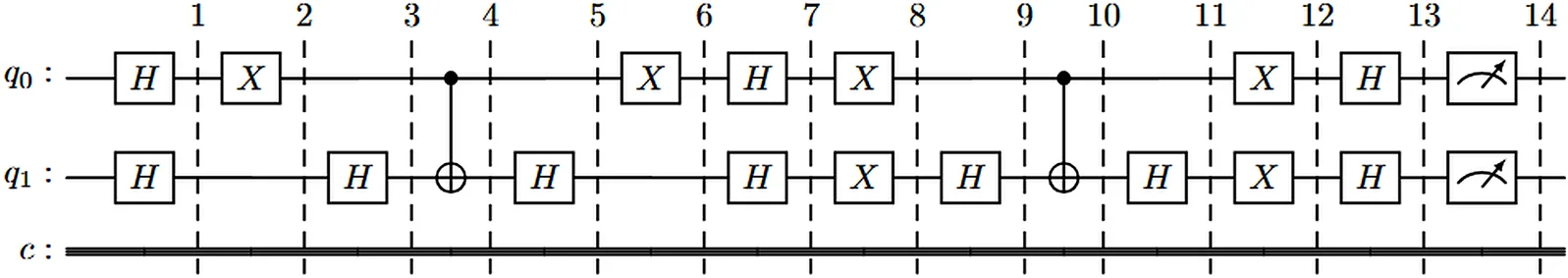
Quantum circuit for 2-qubit Grover’s Search algorithm.

**Fig 28:**
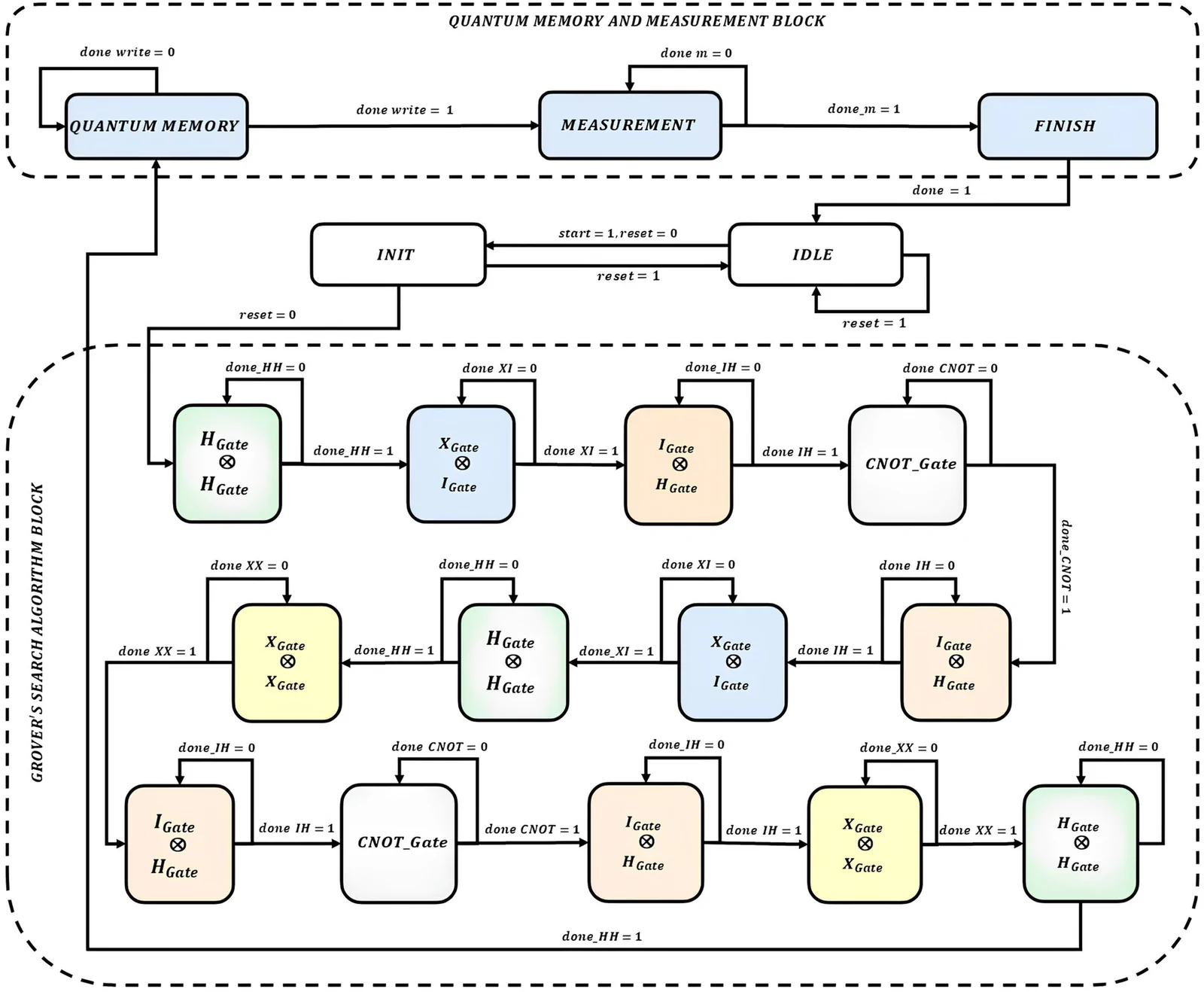
Control-driven execution flow of emulator modules for the 2-qubit Grover’s Search algorithm.

**Fig 29:**
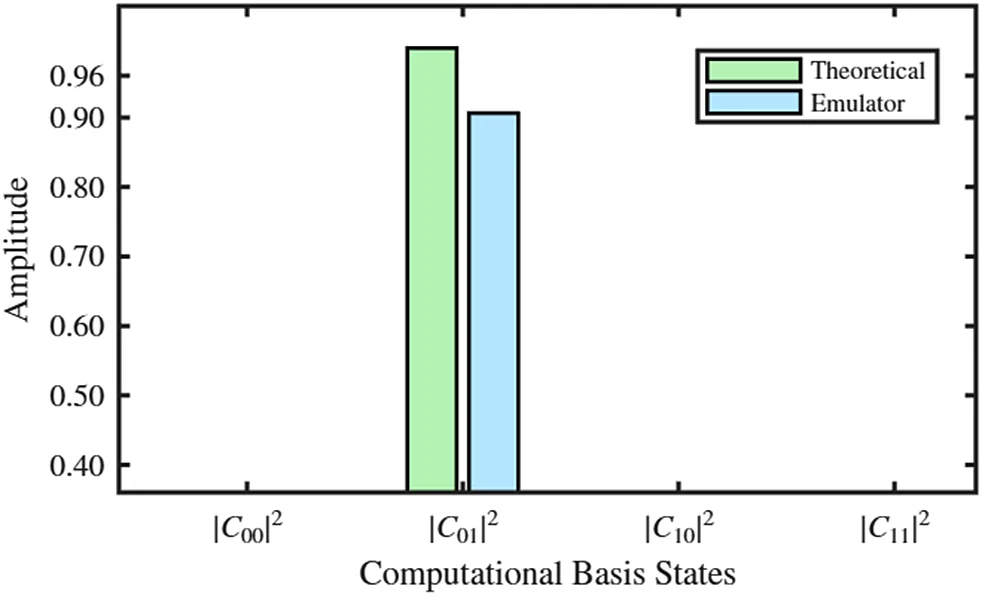
Amplitude comparison of Grover’s Search Algorithm with 2-qubit.

Grover’s Search (GS) algorithm is an unstructured search algorithm that gives the output in $\left[\frac{\pi }{4}\sqrt{N}\right]$ iterations, in contrast to the *N* iterations required by classical systems. The algorithm comprises two parts, i.e., the *Phase-Oracle*, which flips the phase of the marked state, and *the-diffuser*, which amplifies the marked state. Figure 27 presents the circuit for 2-qubit GS. This circuit gives the marked state, i.e., 01, in $\left[\frac{\pi }{4}\sqrt{2^{2}}\right]=1$ iteration instead of 4 = 2^2^ iterations when the oracle is searched classically. Figure 28 presents the execution flow for the given circuit, while Fig 29 represents the measurement results of the emulated circuit (using top down approach), benchmarked against the theoretical output obtained through sequential mathematical analysis of the circuit. In this flow diagram, the transformation of the *H*-CNOT-*H* gate, i.e., the application of the controlled-*Z* gate, is emulated within the at thFSMe behavioral level to ensure a cost-effective implementation.

### 4.5 The BB84 quantum key distribution

BB84 is a communication protocol that provides information theoretically secure key distribution over a quantum channel using a pair of orthogonal bases. For emulation, the selected base pair is *Z* and *Y*. Using bases and keys randomly generated at the sender’s end, the protocol emulation achieves a key-agreement rate of 76.75% at the receiver’s end. Table 9 shows the working of QKD for the distribution of *key* = 01001010.

**Table 9:** Simplified workflow of the *ZY*-BB84 quantum key distribution protocol.

*Bit Number*	7 6 5 4 3 2 1 0
**BB84 Sender Node**	
*Key*	0 1 0 0 1 0 1 0
*Random Basis*	*Z Z Y Y Y Z Y Y*
*Key Qubit*	$|1\rangle \;|0\rangle \;|\dot{\iota }\rangle \;|-\dot{\iota }\rangle \;|\dot{\iota }\rangle \;|0\rangle \;|\dot{\iota }\rangle \;|-\dot{\iota }\rangle$
**BB84 Receiver Node**	
*Key Qubit*	$|1\rangle \;|0\rangle \;|\dot{\iota }\rangle \;|-\dot{\iota }\rangle \;|\dot{\iota }\rangle \;|0\rangle \;|\dot{\iota }\rangle \;|-\dot{\iota }\rangle$
*Random Basis*	*Z Y Y Z Y Y Y Z*
*Key*	0 1 0 1 1 1 1 0

**Fig 30:**
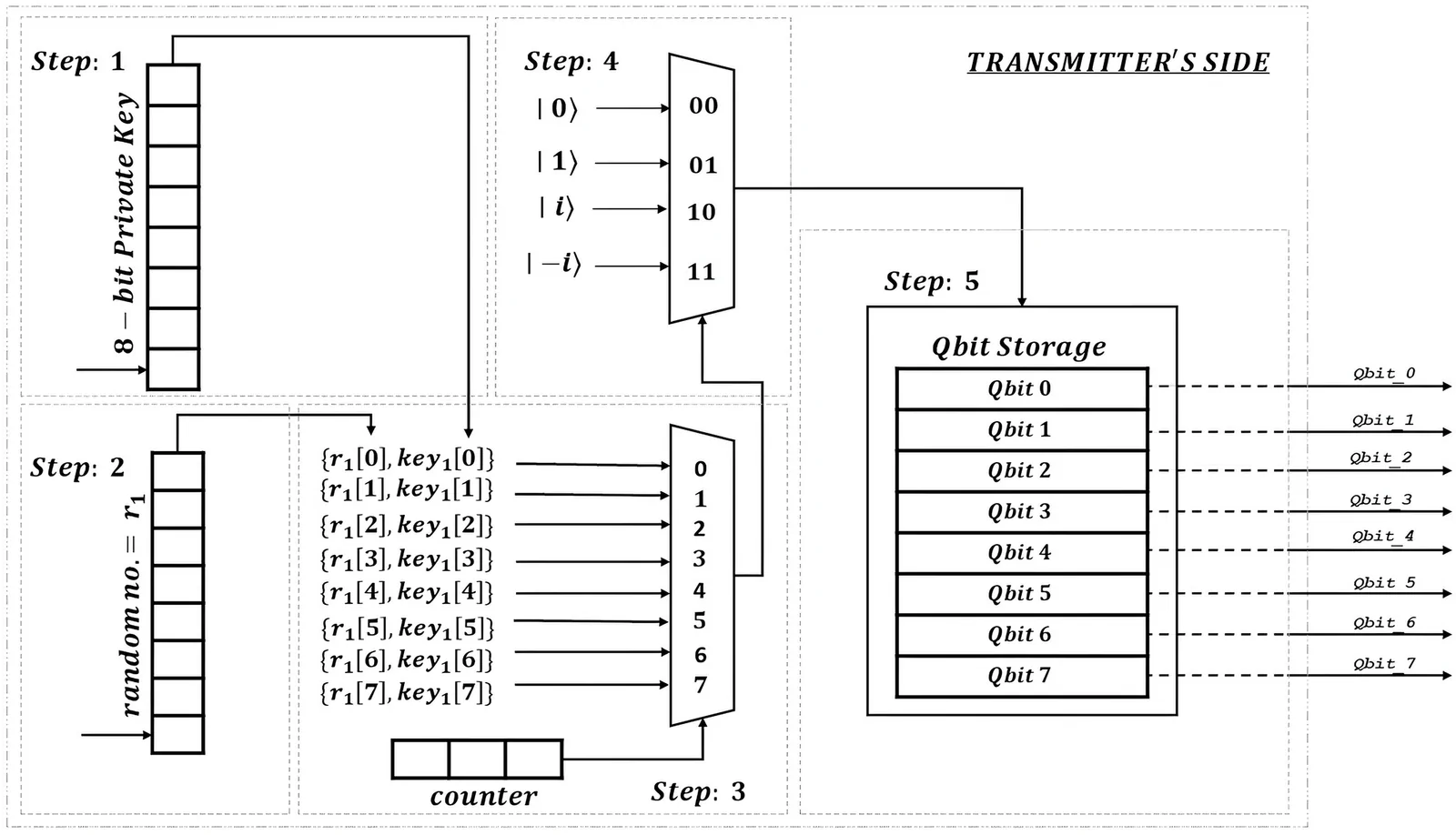
The transmitter emulation for the BB84 protocol.

Since BB84 is a communication protocol, instead of a control flow diagram, the architectures of the sender and receiver modules are designed. Figure 30 presents the sender module, where the key and bases are randomly selected, and, based on the selection, the classical key is transformed into quantum information, bit by bit.

**Fig 31:**
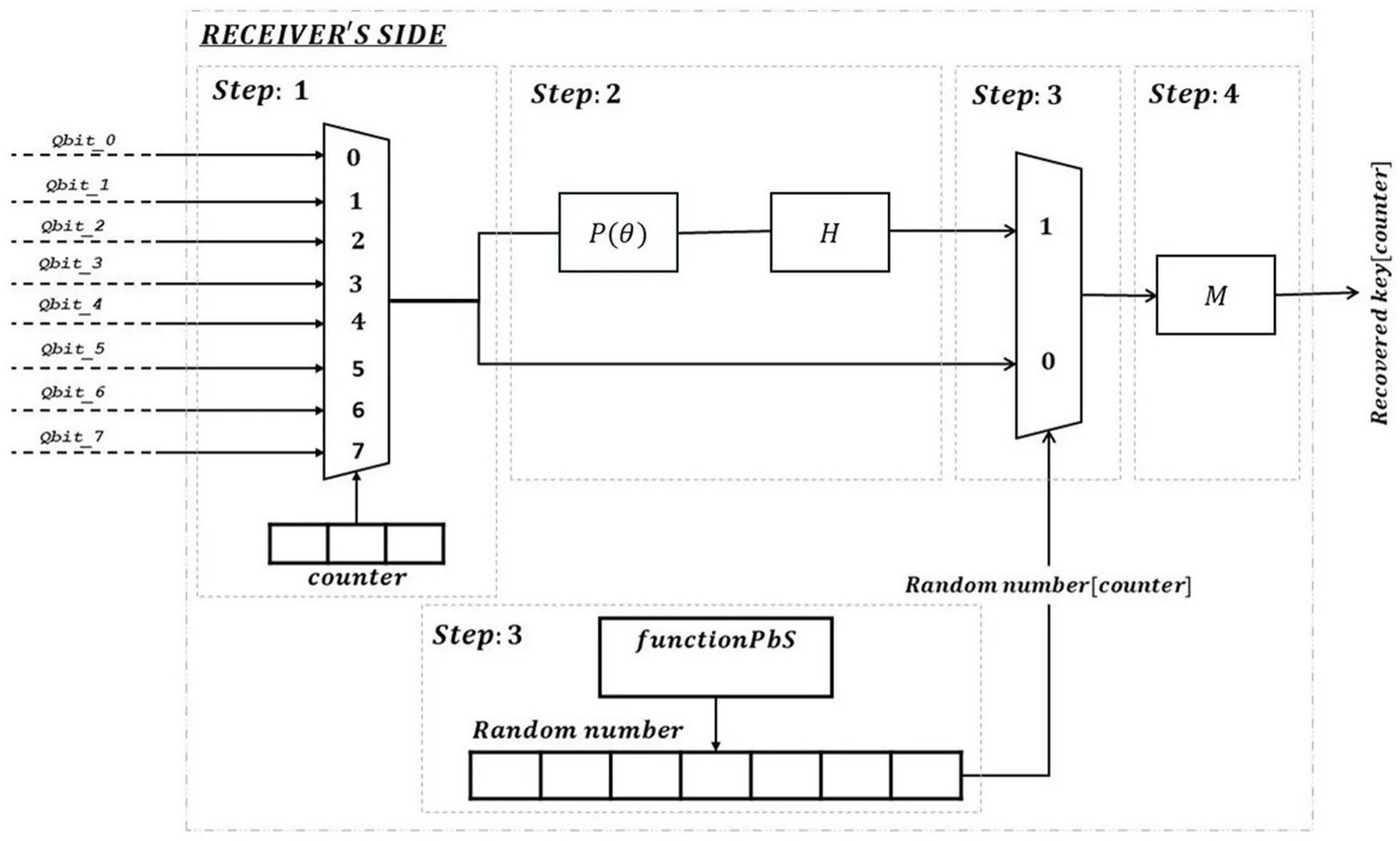
The receiver emulation for the BB84 protocol.

At the receiver’s side in Fig 31, the received quantum information is processed qubit by qubit. Each qubit is measured against randomly selected bases, i.e., *Z* or *Y*, to generate a classical key. Since the proposed emulation is designed for the *Z* basis, the application of the *Phase* gate $\left(\theta =270^{0}\right)$ followed by the *Hadamard* gate replicated the effect of measurement in the *Y* basis.

**Fig 32:**
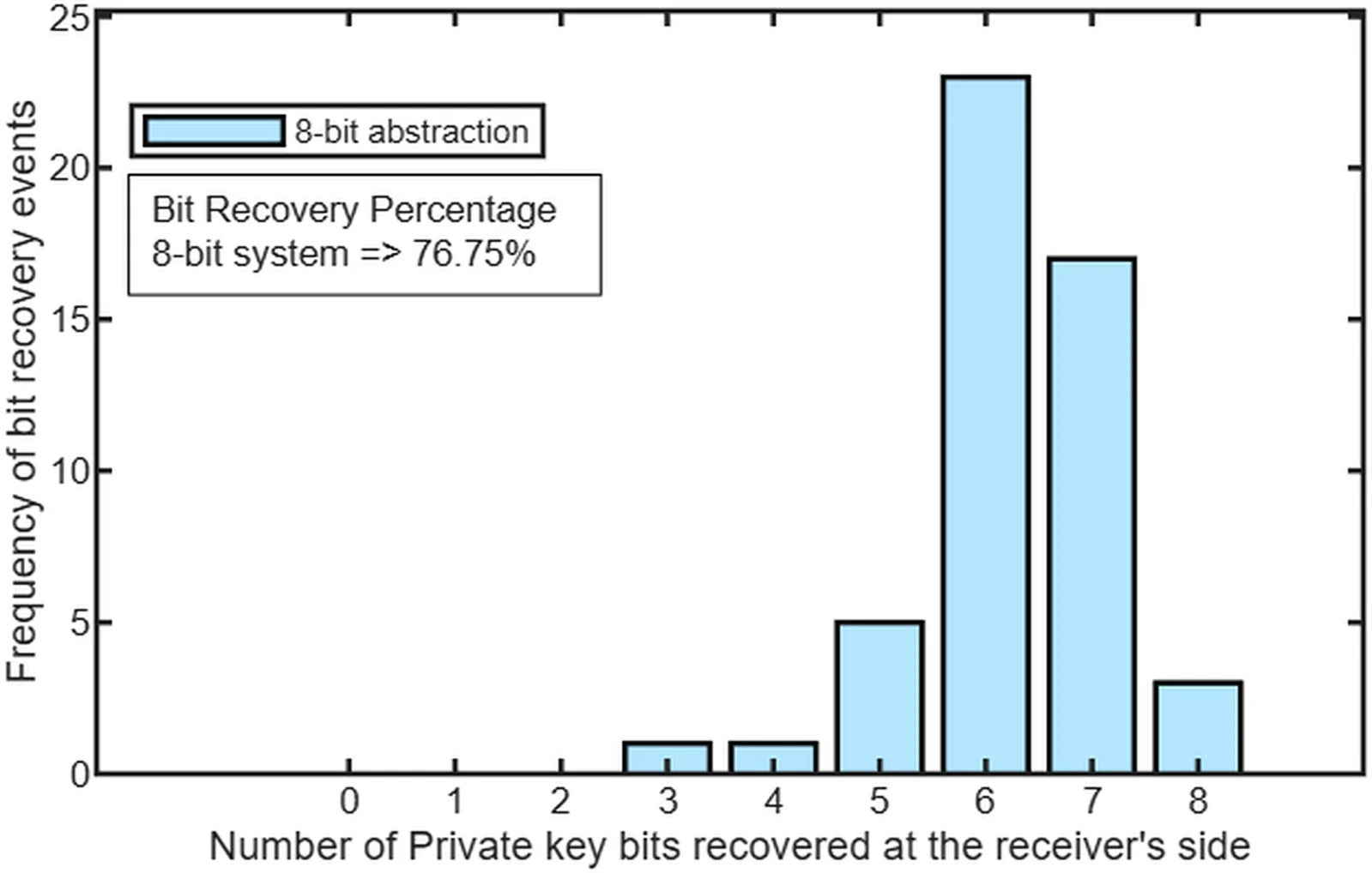
The experimental data for the *BB*84 emulation.

The experiment runs across 100 instances, and Fig 32 shows that the average key agreement rate in the emulated setup is 76.75%, i.e., comparable with the theoretical rate of 75%.

## 5 Performance analysis

The emulator stands out as a highly reliable tool for evaluating the performance of quantum algorithms, ensuring that experimental probabilities align closely with theoretical expectations. The architecture presented in Section 3 uses a Q(2,6) fixed-point representation for quantum emulation. This quantizes the Bloch sphere and introduces truncation error. Thus, to improve emulation performance relative to the theoretical results, the register size may be extended to a Q(2,30) fixed-point representation. This would improve the precision of algebraic calculations, making the system accurate. Table 10 presents a synthesis report on the primary gate’s accuracy and resource utilization on the *Artix-7*, illustrating the inverse relationship between accuracy and implementation cost. Specifically, for unitary gates(*S*, *T* and *Phase*), the Table 10 shows that the emulator effectively reproduces the expected magnitudes of |*C*_0_| and |*C*_1_| for the |0⟩ and |1⟩ states, respectively, with the input |+⟩ and phase angle $\frac{\pi }{3}$ used in *phase* gate. For a 2-qubit system, the result of CNOT with inputs |+⟩ and |−⟩ is as shown in the Table 10. The deviation from the theoretical outcomes is minimal, as indicated by the error rates.

**Table 10:** Comparison table of quantum computing unitary gates.

Theoretical probabilities at Q(2,6)				
**Unitary Gates**	**Error Rate**		**Resource Utilization**	
	|0⟩	|1⟩	**LUT**	**Slice Count**
***T*-Gate**	1%	4%	310	113
***S*-Gate**	1%	1%	313	119
***Phase*-Gate**	1%	8%	323	121
***CNOT*-Gate**	3.1%	3.1%	1921	748
**Theoretical probabilities at Q(2,30)**				
**Unitary Gates**	**Error Rate**		**Resource Utilization**	
	|0⟩	|1⟩	**LUT**	**Slice Count**
***T*-Gate**	0.002%	2%	447	176
***S*-Gate**	0.002%	0.002%	397	147
***Phase*-Gate**	0.002%	4.23%	486	209
***CNOT*-Gate**	2.15%	2.15%	2791	879

The primary contributors to the emulation error are as follows:

1. *Qubit error:* At the input, when a qubit is stored within a register file utilizing a six-bit precision, the superposition states of the basis are subjected to quantization, resulting in an inherent representational error for the qubit. Additionally, the fixed-point representation of qubit flip-flops results in truncation of gate outputs, thereby introducing computational inaccuracies.
2. *Quantum unitary gate error:*The fixed-point (Q(2,6)) gate representation introduces an error due to the presence of irrational numbers within the matrices of unitary gates, including the *Phase*, *S*, *T*, and CNOT gates, which are integral to the quantum circuit design.
3. *Multiplication error*: Multiplication errors occur during gate operations on the FPGA emulator due to rounding and truncation in fixed-point calculations. These errors propagate through subsequent operations, significantly reducing qubit state fidelity.
4. *Measurement error*: Measurement errors in quantum systems can occur because the Pseudo-Random Number Generator (PRNG) used for qubit state measurements produces values with limited precision. Since the PbS function generates eight-bit integer outputs, the discrete nature of the PRNG reduces the accuracy of qubit measurements, leading to quantization errors that impact the precision of the results.

The errors illustrated above demonstrate an inverse relation with register size, as elaborated in the Table 10. The error is quantified as the difference between the emulator outcomes and the corresponding theoretical results, as shown in Eq 33. To rigorously quantify the discrepancy between the theoretically predicted and emulator-obtained amplitudes of a single-qubit state, the error was first assessed by calculating the magnitudes of the resulting coefficients. The magnitude of the emulator amplitude is defined as:


$$\begin{array}{c} |C_{0}|=\sqrt{|\alpha_{0}{|}^{2}+|\hat{\iota }\beta_{0}{|}^{2}},\;|C_{1}|=\sqrt{|\alpha_{1}{|}^{2}+|\hat{\iota }\beta_{1}{|}^{2}} \end{array}$$ (32)

The absolute amplitude deviations are then computed as


$$\begin{array}{c} \Delta |C_{0}|=||C_{0}^{th}|-|C_{0}^{\text{emu}}||,\;\Delta |C_{1}|=||C_{1}^{th}|-|C_{1}^{\text{emu}}|| \end{array}$$ (33)

Finally, the relative percentage errors are calculated according to


$$\begin{array}{ccc} E_{0} & = & \frac{\Delta |C_{0}|}{\left|C_{0}^{\text{th}}\right|}\times 100\%\;\text{for }C_{0}^{\text{th}}>0, \end{array}$$ (34)

$$\begin{array}{ccc} E_{1} & = & \frac{\Delta |C_{1}|}{\left|C_{1}^{\text{th}}\right|}\times 100\%\;\text{for }C_{1}^{\text{th}}>0 \end{array}$$ (35)

Error rate decreases from 3.1% at Q(2,6) to 2.15% at Q(2,30) for the CNOT gate. This improvement in accuracy is associated with higher resource utilization. Look Up Tables (LUTs) and slice counts rise from 1921 to 2791 and 748 to 879, respectively. This reflects a trade-off between precision and computational resources.

Furthermore, the performance of the base operators in the proposed work is benchmarked against a commercial simulator and IBM Quantum Hardware. The comparison circuit comprises qubit initialization, a unitary transformation, and a 100-shot measurement. Table 11 shows that the proposed FPGA emulator achieves significantly lower execution time, primarily due to the inherent parallelism and fine-grained logic control offered by FPGA technology. It should be noted, however, that the FPGA emulator, IBM Aer simulator, and IBM Quantum Hardware represent fundamentally different execution environments. Consequently, the comparison is intended for functional benchmarking rather than a direct hardware-performance evaluation.

**Table 11:** Benchmarking of proposed emulation with commercial setups. Energy values for the IBM Aer simulator represent estimated energy consumption on the CPU during simulation, while values for the proposed emulator are directly measured on the FPGA hardware.

Metric	Proposed emulator	IBM Aer	IBM Hardware
**Real Time execution**	Yes	Yes	Via Cloud
**Deterministic**	High	High	Noise limited
**Hardware**	Artix-7 (FPGA)	Intel (CPU)	IBM_Fez (QPU)
***T-Gate***			
**Accuracy**	91%	95%	100%
**System Latency**	18858 ns	3645500 ns	Cloud queue dependent
**Gate Time**	844.9612 ns	199034.000 ns	Intrinsic duration
**Avg Power**	0.004 W	12.2135 W	Unavailable due to cloud-managed hardware access
**Energy/op**	3.34375 nJ	1017520.00 nJ	
***S-Gate***			
**Accuracy**	91%	98%	96%
**System Latency**	18858 ns	6678300 ns	Cloud queue dependent
**Gate Time**	835.9375 ns	83311.000 ns	Intrinsic duration
**Avg Power**	0.004 W	12.2135 W	Unavailable due to cloud-managed hardware access
**Energy/op**	3.34375 nJ	1017520.00 nJ	
***Phase-Gate***			
**Accuracy**	91%	90%	88%
**System Latency**	18858 ns	7425600 ns	Cloud queue dependent
**Gate Time**	844.9612 ns	68983.000 ns	Intrinsic duration
**Avg Power**	0.005 W	14.9204 W	Unavailable due to cloud-managed hardware access
**Energy/op**	4.22481 nJ	1029250.00 nJ	
***CNOT-Gate***			
**Accuracy**	86%	80%	92%
**System Latency**	18858 ns	3229300 ns	Cloud queue dependent
**Gate Time**	971.4286 ns	113064.000 ns	Intrinsic duration
**Avg Power**	0.024 W	13.8815 W	Unavailable due to cloud-managed hardware access
**Energy/op**	23.31429 nJ	1569510.00 nJ	

The reduction in measurement accuracy observed for the proposed emulator under the given experimental setup is primarily attributable to the limited shot count and the pseudo-random nature of the measurement module, whereas the deviations observed on IBM Quantum Hardware arise from physical noise sources such as gate errors, decoherence, and readout errors. This observation is further supported by the high accuracy of the quantum state representation during unitary transformations, as presented in Table 10.

Table 11 also includes the energy consumption of the proposed FPGA emulator and the IBM Aer simulator to provide additional perspective on the resource-efficient nature of the proposed FPGA implementation. The synthesized gate’s dynamic power was estimated in Vivado and converted to energy per operation, ranging from 3.34375 nJ to 23.31429 nJ for the evaluated gates. For IBM Aer, energy was calculated as the CPU package power measured using Intel Power Gadget multiplied by the execution time (P×T). The FPGA values represent gate-level logic power, whereas the IBM Aer measurements include whole-system CPU overhead, such as operating system and memory activity. Owing to the heterogeneous architectures and fundamentally different measurement methodologies, the two results are not directly comparable. Consequently, the reported energy values are intended only to provide qualitative insight into the resource requirements of the proposed FPGA implementation, rather than to support absolute energy-efficiency comparisons. For IBM Quantum Hardware, per-gate energy is reported as unavailable due to cloud-managed hardware access, since users cannot access the hardware-level power or energy measurements required to estimate individual gate energy.

From the perspective of a quantum circuit, the parameters used to quantify the magnitude of computations are:

1. *Width (W):* The total number of qubits utilized.
2. *Depth (D):* The number of sequential gate layers applied.

The discussion on scalability and error accumulation in a quantum circuit when executed on the emulator is as follows:

1. *Width Analysis:* From an 8-bit emulation viewpoint, each additional qubit introduces extra state-storage overhead. In the proposed implementation, the quantum state associated with one qubit is represented using a register file storing four *Q*(2,6) signed values (32-bits). The current prototype demonstrates accurate emulation of a small quantum instance with width *W* = 2 and depth *D* = 6, i.e., the Deutsch algorithm, validating correct fixed-point gate sequencing and 2-qubit entanglement generation. At this scale, the required state storage remains manageable, since only a limited number of correlated amplitudes must be maintained.

However, as circuit width increases and multi-qubit entanglement becomes more complex, the underlying quantum state can no longer be described with such limited storage. In general, highly entangled systems require tracking $2^{W}$ complex amplitudes, implying that memory and datapath requirements scale exponentially as $O(2^{W})$ with the number of qubits.

Thus, while small-width emulation can be achieved efficiently, scalability beyond a few qubits is fundamentally constrained by the exponential growth of the state space, which represents a primary performance limit for quantum circuit simulators and emulators.

Long-term scalability may be pursued through architectural extensions such as BRAM-based state storage, distributed multi-FPGA partitioning, hybrid FPGA – GPU acceleration, or approximate simulation techniques (e.g., tensor-network or algorithm-specific reduced-state methods). These directions provide a pathway toward supporting larger post-quantum cryptanalytic workloads in future work.

2. *Depth Analysis:* In the emulation architecture, a single increment in the depth refers to the instantiation of a quantum gate emulation circuit, followed by storing the output in a quantum memory block. In this process, the following errors affect the accuracy of the output state probabilities.

- The input is quantized in the form of *Q*(2,6) fixed-point representation of probability states. This limits the superposition states of a qubit.
- Since the unitary operators are expressed as matrices, and every entry of these matrices is expressed in *Q*(2,6) signed fixed-point number, an error is introduced in gate representation.
- Qubit transformations are implemented using matrix multiplication, which involves multiplication followed by addition in fixed-point representation. In a *Q*(2,6) system, multiplying two fixed-point values expands the intermediate result to *Q*(5,12) due to bit growth in both integer and fractional parts. According to the emulation architecture, this *Q*(5,12) result is then truncated back to *Q*(2,6) to maintain the defined fixed-point format as per the emulation architecture.

For demonstration purposes, a phase gate is applied to the state |1⟩ to achieve a complete 2π rotation on the Bloch sphere, i.e., sequential applications of P(π/4) for eight consecutive iterations. Table 12 presents the gate depth analysis of the proposed emulator.

**Table 12:** Gate depth analysis of proposed emulator.

Input	Theoretical o/p	Emulator o/p	Error
(0)|0⟩ + $(1+\hat{\iota }0)|1\rangle$	(0)|0⟩ + $(\frac{1}{\sqrt{2}}+\hat{\iota }\frac{1}{\sqrt{2}})|1\rangle$	(0)|0⟩ + $(0.7071+\hat{\iota }0.7071)|1\rangle$	0.0002
(0)|0⟩ + $(0.7071+\hat{\iota }0.7071)|1\rangle$	(0)|0⟩ + $(0+\hat{\iota }1)|1\rangle$	(0)|0⟩ + $(0.0000+\hat{\iota }0.9687)|1\rangle$	0.0313
(0)|0⟩ + $(0.000+\hat{\iota }0.9687)|1\rangle$	(0)|0⟩ + $(-\frac{1}{\sqrt{2}}+\hat{\iota }\frac{1}{\sqrt{2}})|1\rangle$	(0)|0⟩ + $(-0.6718+\hat{\iota }0.6718)|1\rangle$	0.0532
(0)|0⟩ + $(-0.6875+\hat{\iota }0.6875)|1\rangle$	(0)|0⟩ + $(-1+\hat{\iota }0)|1\rangle$	(0)|0⟩ + $(-0.9375+\hat{\iota }0.0000)|1\rangle$	0.0625
(0)|0⟩ + $(-0.9531+\hat{\iota }0.0000)|1\rangle$	(0)|0⟩ + $(-\frac{1}{\sqrt{2}}-\hat{\iota }\frac{1}{\sqrt{2}})|1\rangle$	(0)|0⟩ + $(-0.6562-\hat{\iota }0.6562)|1\rangle$	0.0722
(0)|0⟩ + $(-0.6562-\hat{\iota }0.6562)|1\rangle$	(0)|0⟩ + $(0-\hat{\iota }1)|1\rangle$	(0)|0⟩ + $(0.0000-\hat{\iota }0.9062)|1\rangle$	0.0938
(0)|0⟩ + $(0.000-\hat{\iota }0.9062)|1\rangle$	(0)|0⟩ + $(\frac{1}{\sqrt{2}}-\hat{\iota }\frac{1}{\sqrt{2}})|1\rangle$	(0)|0⟩ + $(0.6250-\hat{\iota }0.6250)|1\rangle$	0.116
(0)|0⟩ + $(0.6250-\hat{\iota }0.6250)|1\rangle$	(0)|0⟩ + $(1+\hat{\iota }0)|1\rangle$	(0)|0⟩ + $(0.8750+\hat{\iota }0.0000)|1\rangle$	0.125

The depth analysis indicates that truncation-induced error accumulates approximately linearly with circuit depth in the *Q*(2,6) fixed-point representation. After eight consecutive *phase* operations, the observed amplitude deviation reaches approximately 0.125, which closely corresponds to $8\times 2^{-6}$. The term 2^-6^ arises from the fractional precision of the *Q*(2,6) format, where six bits are allocated to the fractional part, yielding a resolution of 2^-6^ = 0.015625.

This behaviour confirms that the dominant source of inaccuracy originates from repeated fixed-point truncation, leading to cumulative error proportional to the number of gate applications. Consequently, while the *Q*(2,6) representation is suitable for shallow circuits, deeper quantum circuits benefit from higher-precision formats such as *Q*(2,30) to mitigate error growth.

The Bell-states creation and the Deutsch algorithm with *f*(*x*) are executed on all three setups. Table 13 presents the performance of these setups compared to the theoretical ground truth. The IBM QC (ibm−aachen) platform exhibits noise, instability, and error rates of $7.10\times 10^{-4}$ for 2*Q* (best), $6.50\times 10^{-3}$ for 2*Q* (layered), $1.872\times 10^{-3}$ for *CZ*, and $2.114\times 10^{-4}$ for *SX*. These errors stem from decoherence and noise, directly impacting its performance [25].

**Table 13:** Comparison of Quantum Computing Algorithms Based on Error Rate and FPGA Resource Utilization.

Experimental Probabilities of Bell States					
**Bell States**	**Error Rate**			**Resource Utilization**	
	**EMU**	**AER**	**IBM**	**LUT**	**Slice Count**
$|\Phi^{+}\rangle$	2%	1%	6%	699	235
$|\Psi^{+}\rangle$	2%	3%	2%	718	285
$|\Phi^{-}\rangle$	2%	2%	6%	695	269
$|\Psi^{-}\rangle$	2%	3%	2%	725	287
**Experimental Probabilities of the Deutsch Algorithm**					
**Phase Oracle**	**Error Rate**			**Resource Utilization**	
	**EMU**	**AER**	**IBM**	**LUT**	**Slice Count**
$Xnor_{f}$	14%	16%	22%	2311	800
**Superdense Coding Quantum Circuit (16-qubit Circuit)**					
**Communicated Classical Information**	**Error Rate**			**Resource Utilization**	
	**EMU**	**AER**	**IBM**	**LUT**	**Slice Count**
0*x*781*B*	4%	0%	100%	589	424
**Grover’s Search Algorithm (2-qubit Circuit)**					
**Marked State**	**Error Rate**			**Resource Utilization**	
	**EMU**	**AER**	**IBM**	**LUT**	**Slice Count**
01	7%	0%	9%	3977	3410

Similarly, the Superdense coding and Grover Search algorithms were executed and evaluated across all three setups. The IBM QC platform (*ibm_fez*) exhibits noise, instability, and error rates of $1.23\times 10^{-3}$ for 2Q (best), $4.88\times 10^{-3}$ for 2Q (layered), $2.657\times 10^{-3}$ for CZ, and $3.117\times 10^{-4}$ for SX, which adversely affect its performance during quantum computations [26]. The proposed emulator is independent of environmental noise and cannot, thus, be compared one-to-one with the actual quantum computer. The IBM simulator, on the other hand, uses a state-space representation of qubits; thus, it can serve as a valid benchmark for the proposed work.

Table 13 shows that the Q(2,6) emulation architecture produces results comparable to those of the open-source IBM simulator. Therefore, the proposed Q(2,6) emulation architecture is a standalone framework that can execute quantum circuits and produce results comparable to those of a globally recognized open-source simulator, i.e., the *IBM-Aer* simulator. Table 13 provides a comprehensive overview of the accuracy and resource utilization of quantum computing algorithms. It underscores the emulator’s proficiency in replicating the expected probabilities of *Bell-States*, with square magnitudes $|C_{00}{|}^{2}$, $|C_{01}{|}^{2}$, $|C_{10}{|}^{2}$, and $|C_{11}{|}^{2}$, using inputs |0⟩ and |0⟩ for both qubits. The deviation from theoretical results remains minimal, with the emulator’s average error rate at 2%, compared to 2.25% for the IBM simulator and 3.5% for *IBM-QC*. This precision extends to the Deutsch algorithm with the *Xnor* function, where the emulator achieves an error rate of 14%, compared to 16% for *IBM-Aer* and 22% for *IBM-QC*.

The Superdense Coding protocol was scaled by executing eight independent 2-qubit circuits in parallel, enabling the transmission of a 16-bit binary word (0*x*781*B*). The *IBM-Aer* simulator provides ideal, noise-free state-vector simulation and therefore achieves near-perfect accuracy. The proposed FPGA emulator exhibits a small reduction in accuracy due to finite precision arithmetic and measurement sampling effects. In contrast, IBM quantum hardware is subject to physical noise sources, including gate errors, decoherence, and readout errors, which contribute to greater deviations from the theoretical result, particularly when a limited number of measurement shots are used, as reported in Table 13. The resource utilization results indicate that sufficient LUT resources remain available on the *Artix-7* FPGA, suggesting the potential to further scale the same Superdense coding implementation to support larger message widths.

The Grover Search algorithm was implemented as a 2-qubit circuit targeting the marked state $|q_{0}q_{1}\rangle =|01\rangle$. The *IBM-Aer* simulator achieved the ideal noise-free result with a 0% error rate. The proposed FPGA emulator exhibited an error rate of 7%, primarily due to finite-precision arithmetic and measurement sampling effects, while utilizing 3977 LUTs and 3410 slices, as reported in Table 13. In contrast, the IBM quantum hardware produced an error rate of 9%, which can be attributed to physical noise sources such as gate errors, decoherence, and readout errors. Consequently, the FPGA emulator demonstrates performance that is closer to the theoretical ground truth while providing a resource-efficient platform for quantum algorithm emulation.

Two key features that determine the completeness and fidelity of a quantum emulation architecture are the inclusion of a measurement module and validation against a standard reference simulator. Architectures that incorporate both features can be regarded as complete in functionality and reliable in accuracy.

Table 14 presents a comparative analysis of the proposed system with the emulator available in the literature based on the metrics, i.e., the size and complexity of the quantum circuits used for functional verification, the target FPGA platform, hardware resource utilization, and the numerical precision employed for the classical representation of quantum states.

**Table 14:** Comparative assessment of the proposed emulator and existing FPGA-based quantum emulation architectures.

Emulators	Nqubits	Numeric Representation	Benchmark	Measurement Emulation	Logic Utilization	Board	Functional Verification	
							Random Circuit	Specialized Algorithm
[27] (2018)	2−qubit	fixed-point	×	✓	8K ALMs (Deutsch algorithm emulation)	Altera Cyclone V	×	Deutsch Algorithm
[28] (2024)	7−qubit	fixed-point	QuEst	✓	168,931 ALMs (7−qubit circuit)	Intel Agilex F-Series	✓	–
[14] (2024)	2−qubit	16−bit fixed-point	Matlab	×	31,305 ALMs (2−qubit emulation architecture)	Arria 10	✓	–
[16] (2025)	5−qubit	20−bit fixed-point	MQT Bench	×	20,993 LE (5−qubit emulation architecture)	Intel Cyclone 10LP	✓	3−qubit QNN, Quantum Teleportation
[15] (2024)	16−qubit	20−bit fixed-point	Qiskit	×	7751 CLB (16−qubit emulation architecture)	AMD Kria KV260 SoM	✓	–
Proposed emulator	8×2−qubit	8−bit, 32−bit fixed-point	Qiskit, IBM Quantum Computer	✓	589 LUTs (8×2−qubit Superdense coding circuit)	Artix−7	✓	Bell States, Deutsch Algorithm, Superdense Coding, Grover Search, BB84 QKD

The target boards for emulations [27,28] and [14] are DSP-enabled and are equipped with high on-chip memory. This gives the proposed design the ability to emulate large quantum circuits at the cost of the hardware. In contrast to that, the proposed emulator and the architectures defined in [16] and [15] are executed on resource-constrained boards with limited memory.

Despite the limited hardware resources, the proposed emulator incorporates a measurement module along with a pseudo-random number generator (PRNG) and demonstrates functional verification across a diverse set of quantum algorithms, including the parallel execution of eight independent 2-qubit Superdense Coding circuits defined in Section 4.3 for the communication of a 16-bit binary word and a Grover’s Search with the circuit depth of 11. Furthermore, the resource utilization analysis indicates that sufficient FPGA resources remain available after implementing the parallel Superdense Coding modules, suggesting that additional independent 2-qubit modules can be instantiated on the target *Artix-7* platform. These results demonstrate the scalability of the proposed architecture through modular replication of the underlying 2-qubit emulation framework.

This enhanced performance is attributable to the emulator’s efficient gate transformations. The process entails transforming input qubits with specified gates, followed by measurement and storage in qubit memory. A base-function cost analysis reinforces the emulator’s resource-efficient performance. Table 15 presents a comparative analysis of the proposed work with the baseline framework in order to quantify the proposed improvements.

**Table 15:** Comparative analysis with the baseline framework.

	[12]	Proposed Work
No. of qubits	1	2
Quantum Gate	*Pauli X*, *Y*, *Z*, *H*	*S*, *T*, *Phase*, CNOT
Board	Spartan-3	Artix-7
Algorithms	BB84	Deutsch, Bell states
Benchmark	Theoretical	Theoretical, IBM-AER simulator, IBM quantum computer
Gate depth analysis	×	✓
Resources (Slice count)	Pauli-X/Y/Z: ~208 slices; *Hadamard*: 256 slices	*S*, *T*, *Phase* Gate: ~113−121 slices; CNOT: 748 slices

The analysis shows that the proposed solution offers a universal set for quantum computation on *Artix-7*, in contrast to superposition emulation on the relatively older board, i.e., *Spartan-3*, used in the baseline framework. This universality enables the emulation of a greater set of quantum circuits in a resource-efficient manner. In addition, the performance evaluation metrics, including gate-depth sensitivity analysis and functional benchmarking against the IBM platform, provide quantitative evidence of the accuracy, robustness, and practical reliability of the proposed FPGA-based quantum emulation framework.

## 6 Conclusions

In conclusion, the proposal for an FPGA-based emulator implemented on the *Artix-7* board using a behavioral model approach offers a cost-effective, scalable platform. It efficiently simulates quantum unitary gates, yielding results comparable to theoretical outcomes. The proposed architecture is designed for *Q*(2,6) and *Q*(2,30) fixed-point data representation, demonstrating the tradeoff between computational resources and the emulator’s efficiency. To validate its performance, Bell states, Deutsch algorithm (for balanced function), Superdense coding, Grover’s search, and BB84 QKD were successfully emulated on a *Q*(2,6) architecture, yielding average error rates of 2%, 14%, 4%, and 7% respectively. The results demonstrate that the proposed emulator provides functionality comparable to that of the *IBM-Aer* simulator while offering improved resource efficiency, scalability, and hardware realizability. These characteristics make it a practical framework for verifying and testing quantum algorithms on FPGA platforms.

## References

[1] PsiQuantum team. A manufacturable platform for photonic quantum computing. Nature. 2025;641(8064):876–83. doi: 10.1038/s41586-025-08820-7 40010377 PMC12095036

[2] Loss D, DiVincenzo DP. Quantum computation with quantum dots. Phys Rev A. 1998;57(1):120–6. doi: 10.1103/physreva.57.120

[3] Bravyi S, Dial O, Gambetta JM, Gil D, Nazario Z. The future of quantum computing with superconducting qubits. J Appl Phys. 2022;132(16). doi: 10.1063/5.0082975

[4] Burkard G, Ladd TD, Pan A, Nichol JM, Petta JR. Semiconductor spin qubits. Rev Mod Phys. 2023;95(2). doi: 10.1103/revmodphys.95.025003

[5] Cirac J, Zoller P. Quantum computations with cold trapped ions. Phys Rev Lett. 1995;74(20):4091–4. doi: 10.1103/PhysRevLett.74.4091 10058410

[6] Computing Q. Top applications of quantum computing for machine learning; 2023. Available from: https://www.quera.com/blog-posts/applications-of-quantum-computing-for-machine-learning

[7] Wang Z, Wang F, Li L, Wang Z, van der Laan T, Leon RCC, et al. Quantum kernel learning for small dataset modeling in semiconductor fabrication: application to ohmic contact. Adv Sci (Weinh). 2025;12(35):e06213. doi: 10.1002/advs.202506213 40548955 PMC12462921

[8] Wang Z, van der Laan T, Usman M. Self-adaptive quantum kernel principal component analysis for compact readout of chemiresistive sensor arrays. Adv Sci (Weinh). 2025;12(15):e2411573. doi: 10.1002/advs.202411573 39854057 PMC12005759

[9] Yole G. Quantum technologies: the market is growing. 2021. Available from: https://www.yolegroup.com/press-release/quantum-technologies-the-market-is-growing/

[10] Hussain AH, Hasan MN, Prince NU, Islam MM, Islam S, Hasan SK. Enhancing cyber security using quantum computing and artificial intelligence: a. PLOS ONE. 2021;15:123–30. doi: 10.1371/journal.pone.0256789

[11] Moawad Y. Architectures and optimisations for FPGA-based simulation of quantum circuits; 2025.

[12] Khalid M, Mujahid U, Jafri A, Choi H, Muhammad N ul I. An FPGA-based hardware abstraction of quantum computing systems. J Comput Electron. 2021;20(5):2001–18. doi: 10.1007/s10825-021-01765-w

[13] Kaye P, Laflamme R, Mosca M. An introduction to quantum computing. 2007.

[14] Giorgio A. Project and implementation of a quantum logic gate emulator on FPGA using a model-based design approach. IEEE Access. 2024;12:41317–53. doi: 10.1109/access.2024.3377458

[15] Conti C, Volpe D, Graziano M, Zamboni M, Turvani G. AMARETTO: enabling efficient quantum algorithm emulation on low-tier FPGAs. arXiv. 2024. doi: arXiv:241109320

[16] Lagostina L, Volpe D, Zamboni M, Turvani G. AEQUAM: accelerating quantum algorithm validation through FPGA-based emulation. IEEE Access. 2025;13:130232–55. doi: 10.1109/access.2025.3589746

[17] Nedjah N, Raposo S, de Macedo Mourelle L. Dedicated hardware design for efficient quantum computations using classical logic gates. J Supercomput. 2023;80(5):7028–70. doi: 10.1007/s11227-023-05687-1

[18] Waidyasooriya HM, Oshiyama H, Kurebayashi Y, Hariyama M, Ohzeki M. A scalable emulator for quantum fourier transform using multiple-FPGAs with high-bandwidth-memory. IEEE Access. 2022;10:65103–17. doi: 10.1109/access.2022.3183993

[19] El-Araby E, Mahmud N, Jeng MJ, MacGillivray A, Chaudhary M, Nobel MdAI, et al. Towards complete and scalable emulation of quantum algorithms on high-performance reconfigurable computers. IEEE Trans Comput. 2023;72(8):2350–64. doi: 10.1109/tc.2023.3248276

[20] Choi S, Lee W. Developing a Grover’s quantum algorithm emulator on standalone FPGAs: optimization and implementation. Quantum Comput J. 2024;15:123–30. doi: 10.1234/qc.2024.01234

[21] Mahmud N, Haase-Divine B, Kuhnke A, Rai A, MacGillivray A, El-Araby E. Efficient Computation techniques and hardware architectures for unitary transformations in support of quantum algorithm emulation. J Sign Process Syst. 2020;92(9):1017–37. doi: 10.1007/s11265-020-01569-4

[22] Mujahid U, Khalid M, Najam-ul-Islam M. FPGA based emulation of B92 QKD protocol. In: 2023 57th Annual Conference on Information Sciences and Systems (CISS), 2023. 1–5. 10.1109/ciss56502.2023.10089628

[23] Zahra A, Khalid M. FPGA-based emulation framework for two-qubit quantum computation: design, implementation, and validation. 2026. Available from: 10.17504/protocols.io.dm6gp4bqpgzp/v2 PMC13502598 42636276

[24] Zahra A. FPGA-based emulation framework for two-qubit quantum computation. 2026. Available from: https://github.com/Arfeen983/FPGA-Based-Emulation-Framework-for-Two-Qubit-Quantum-Computation 10.1371/journal.pone.0356172 PMC13502598 42636276

[25] IBM Quantum. Compute resources — ibm_aachen QPU. Available from: https://eu-de.quantum.cloud.ibm.com/computers?system=ibm_aachen. 2025.

[26] IBM Quantum. Compute resources: IBM Fez. IBM Quantum. Available from: https://quantum.cloud.ibm.com/computers?system=ibm_fez. 2026.

[27] Pilch J, Długopolski J. An FPGA-based real quantum computer emulator. J Comput Electron. 2018;18(1):329–42. doi: 10.1007/s10825-018-1287-5

[28] Belfore I, Lee A. A scalable FPGA architecture for quantum computing simulation. arXiv. 2024. https://arxiv.org/abs/240706415

