# Supplementary material for: FPGA-based emulation framework for two-qubit quantum computation: Design, implementation, and validation
Source: PLoS One. 2026 Aug 24;21(8):e0356172. doi: 10.1371/journal.pone.0356172 (PMC13502598; doi:10.1371/journal.pone.0356172)
Supplement: Supplementary file 1 [file pone.0356172.s001.pdf]

Respected editor,

I hope you are fine. Firstly, we thank the anonymous reviewers for their valuable comments and feedback, which will ultimately enhance the paper's overall quality. The following are the reviewer's comments and the actions taken to improve the paper in response to the valuable suggestions.

Comments of Reviewer 1:

**S. No.**	**Reviewer's Comments**	**Author (s) response**	**References**
1.	Fig. 10 contains very small descriptions, unfortunately, due to the fact that it is a bitmap, enlarging the image does not help; we still have blurry text.	Figure 10, now referred to as Fig. 11, has been updated to present the precise FSM for quantum gate emulation.<br>The quality of all the figures presented in the paper is upgraded	Figure 11<br><br>All the figures in the paper
2.	1. The authors did not describe the details of the implementation of qubit emulation (how many LUTs, BRAM and etc.),<br>2. nor did they present the hardware platform on which the emulation is carried out, only in the summary it was written that the Zynq-7000 was used,	The response to this question comprises two parts:<br><br>1. Section 3 has been revised to include detailed resource utilisation reports for each proposed module, including LUT, flip-flop, Slice count, power, and latency. It is important to note that Block RAM (BRAM) resources were intentionally not employed in the presented architectures. This design choice stems from the limited state-space size of the targeted single- and two-qubit systems, where amplitude storage requirements are minimal. Under these conditions, register-based storage provides single-cycle deterministic access and enables efficient amplitude permutation without introducing BRAM access latency or underutilised memory blocks.<br>2. We thank the reviewer for pointing out the ambiguity regarding the hardware platform. In the original manuscript, the Zynq-7000 SoC was mentioned; however, the proposed architectures utilise only the programmable logic (PL) fabric and do not rely on the embedded processing system (PS). Upon careful	1. Section 3.3 (Table 5-8)<br>2. Throughout the paper

		consideration, we recognise that referencing the SoC platform was not the most appropriate choice, as the design targets a pure programmable-logic implementation.<br><br>Accordingly, the hardware platform has been reselected to a standalone Artix-7 FPGA device, which more accurately reflects the architectural intent of the work. The manuscript has been revised consistently to state the Artix-7 device explicitly, and all synthesis results and resource utilisation reports have been regenerated for this target platform.<br><br>This correction eliminates potential confusion between SoC-based and FPGA-only implementations and ensures that the reported results accurately correspond to a programmable logic–only realisation.	
3.	1. how the probability amplitudes were represented, with what accuracy, how the calculations were performed,<br>2. how quantum noise was simulated,	The response to this question comprises two parts:<br><br>1. The manuscript has been revised to detail the probability-amplitude representation and the numerical accuracy explicitly. Each amplitude is represented in the signed fixed-point format $Q(2, m)$, with the real and imaginary components stored separately. Although the theoretical dynamic range $Q(2, m)$ is $[-2, 2-2^{-m}]$, the effective operational range is restricted $[-1, 1]$ due to quantum-state normalisation constraints with the resolution of 2^{-m}. The proposed design follows the baseline framework's representation model to ensure consistency and comparability.	1. Section 2,3<br>2. Section 1

		The architecture is designed for $Q(2,6)$ signed fixed-point representation. Additionally, a cost–accuracy analysis has been included by scaling the architecture to a 32-bit $Q(2,30)$ configuration, and the corresponding performance results are presented in the Performance Analysis section.<br><br>2. As stated in the Introduction, the proposed framework is designed as a classical, controlled emulation platform intended for validating quantum gate operations under ideal unitary evolution. Accordingly, quantum noise effects such as decoherence, depolarisation, or amplitude damping are not modelled in this work. The architecture operates purely on state-vector representation and implements deterministic unitary transformations followed by probabilistic measurement. Modelling quantum noise would require non-unitary evolution using density-matrix formalism and quantum channel operators, which is beyond the scope of the present FPGA-based emulator.	
4.	1. how measurements were conducted, the expectation value, and other types of measurements,<br>2. Quantum channels have been simulated,	1. The measurement subsection has been expanded to clearly explain how measurements are performed in the proposed emulator. We now describe the multi-shot probabilistic sampling process based on the Born rule for both single- and two-qubit cases. The PRNG-based interval selection method is detailed, and it is clarified that each experiment is executed with 100 shots to obtain statistically stable results. We also explain how measurement statistics are derived from the observed frequencies.<br>2. The proposed framework is a computing emulator designed to model ideal unitary evolution of quantum states within a classical FPGA-based architecture. It focuses	1. Section 3.2, Table 11<br>2. –

		exclusively on gate-level state-vector transformations and probabilistic measurement. Physical communication channels, transmission effects, or noise processes associated with quantum communication systems are not modelled in this work, as the objective is to validate computational functionality rather than to simulate the physical-layer behaviour of quantum channels.	
5.	the architecture from Fig. 6, 8 is only a record of the operation of a unitary operation, unfortunately Fig. 6 is misrepresented, because the notation suggests that these are gates controlled by a zero qubit in state one, unless the Authors introduce gates separately for individual qubits, but the graphic form is not unambiguously presented in this case, it is enough to write it directly using Dirac notation and the product of the tensor,	The previously included abstract gate-level representations in Fig. 6 and Fig. 8 have been replaced with detailed FPGA microarchitecture diagrams defined in Fig. 6-9. The updated figures now explicitly illustrate the hardware-level implementation of the corresponding unitary operations and are consistently labelled in accordance with Eqs. (17), (21), (24), and (27). This revision removes the earlier ambiguity in graphical notation and ensures direct alignment between the mathematical Dirac-form expressions and their hardware realisation.	Figure 6-9
6.	the automata shown in Fig. 10, 12, 14 are not properly defined automata, they are a graphical record of the use of individual quantum gates,	1. The concern regarding the definition of automata has been addressed. Figure 10 (now referred to as Figure 11) has been revised to present a formally defined Finite State Machine (FSM) describing the control flow of quantum gate emulation. The updated diagram explicitly specifies the states, transitions, and control logic governing gate execution.<br>2. Figures 12 and 14 (now Figures 15 and 17) have been clarified to represent the operational sequencing of emulator functions rather than formal automata. These diagrams illustrate the order in which the emulator modules are instantiated and executed during circuit evaluation. The captions have been revised to	1. Figure 11<br>2. Figure 15,17

		remove ambiguity and accurately reflect their functional intent.	
	Fig. 11 is not a representation of architecture, but quantum circuits forming a Bell pair,	Thankyou for the kind feedback.<br><br>The caption of Figure 11, now referred to as Figure 14, has been updated to “Quantum circuit for Bell states.”	Figure 14
8.	The authors state that their solution is universal, unfortunately it is not, because in order for it to be simulated, any unitary single gate should be simulated, and such a computational procedure is not presented in the article,	We thank the reviewer for the observation regarding the need to demonstrate universal simulation. To address this, we have added Section 3.5, which elaborates on how our phase-gate implementation serves as a basis for any arbitrary single-qubit unitary. By explicitly mapping the full range of rotations to the parameters (θ, ϕ, λ), we demonstrate that our proposed solution satisfies the criteria for universal single-qubit simulation. We believe this addition clarifies the completeness of the computational procedure.	Section 3.5
	It is possible to point out many other shortcomings, e.g. the lack of analysis of the latency in FPGA calculations,	Thankyou for the kind feedback.<br><br>The latency and power consumption, along with a detailed resource utilisation summary of the proposed operators, are now added as tables 5,6,7,8	Section 3.3.1,Table 5,6,7<br>Section 3.3.2, Table 8
10.	it requires, in my opinion, a complete rewrite.	Thankyou for the kind feedback. The experiments have been extended and reperformed, the figures and tables have been updated, and the description of the proposed work has been carefully reviewed in light of the revisions.	Complete Paper

Comments of Reviewer 2:

S. No.	Reviewer’s Comments	Author (s) response	References
1.	Please add Scalability Discussion: As your approach is very limited to small number of qubit, please clearly state its limitation and discuss how it could be scaled in the long term.	We have added a dedicated scalability discussion in the Performance section, clarifying that the current prototype demonstrates correct two-qubit entanglement emulation ($W = 2$, $D = 6$) with manageable resource usage. We further explain that as circuit width and multi-qubit entanglement	Section 5

		increase, the required state representation grows exponentially, imposing a fundamental limitation on simulators/emulators. Finally, we outline potential long-term scaling directions, including BRAM-based storage, multi-FPGA partitioning, and approximate reduced-state techniques.	
2.	Add experiments to benchmark with other FPGA based emulator, eg, FPGA (ISE) Khalid et al. [10], 2021, listed in table; also consider other benchmarks to add to justify its advantages.	Thankyou for the valuable feedback. The relevant benchmark is added as Table 12.	Section 5, Table 12
3.	Consider gate-depth sensitivity experiments -- The goal is to evaluate how numerical errors accumulate as the depth of the quantum circuit increases. So that the readers could know quantitively the limitation of this approach and when it outforms.	A gate-depth sensitivity analysis has been added to quantify the accumulation of numerical errors in the proposed fixed-point architecture. The study demonstrates that truncation-induced error grows approximately linearly with circuit depth, consistent with the fractional resolution 2^{-6} of the $Q(2,6)$ representation. The results explicitly show that cumulative amplitude deviation after eight consecutive gate applications approaches $8\times2^{-6} = 0.125$, thereby clarifying the practical depth limitations of the 8-bit configuration. This analysis provides readers with a quantitative understanding of accuracy–depth tradeoffs and justifies the inclusion of higher-precision configurations (e.g., $Q(2,30)$) for deeper circuits.	Section 5, Table 10
