# Supplementary material for: FPGA-based emulation framework for two-qubit quantum computation: Design, implementation, and validation
Source: PLoS One. 2026 Aug 24;21(8):e0356172. doi: 10.1371/journal.pone.0356172 (PMC13502598; doi:10.1371/journal.pone.0356172)
Supplement: Supplementary file 2 [file pone.0356172.s002.pdf]

Respected editor,

I hope you are fine. Firstly, we would like to thank the anonymous reviewers for their valuable comments and feedback, which will ultimately enhance the paper's overall quality. The following are the reviewer's comments and the actions taken to improve the paper in response to the valuable suggestions.

Comment of Editor:

S.No.	Editor's Comments	Author (s) response	References
1	According to the reviewers, please only claim with strong evidence e.g., novelty and advancement.	We thank the editor for the suggestion. In response, we have revised the manuscript to avoid overstating novelty claims and clarified that the proposed work is an advancement and extension of the framework presented in the existing literature [1]. The revised text now emphasizes extending the emulator by incorporating a universal quantum gate set, including parameterized Phase gates and the CNOT gate, rather than presenting the work as a completely novel architecture.	Complete Paper, especially Abstract

Comment of Reviewer 1:

S.No.	Reviewer's Comments	Author (s) response	References
1	The authors made a number of corrections and answered the questions and comments posed. The current version can be rated better than the original version of the article. The current figure contains useful information for people who would like to try to implement quantum computing simulation using FPGAs.	We thank the reviewer for the encouraging feedback.	–

Comment of Reviewer 2:

S.No.	Reviewer's Comments	Author (s) response	References
1	The evaluation only includes Bell states and the Deutsch algorithm , which are basic two-qubit demonstrations. There is no validation on more complex quantum algorithms or larger qubit systems, making it unclear whether the framework can scale beyond small test cases.	We thank the reviewer for this valuable observation. In response, we have expanded the validation section by incorporating three additional quantum protocols with varying circuit characteristics.<br>1. Superdense Coding has been scaled to a circuit width of 16 qubits, demonstrating scalability to larger systems.<br>2. Grover's Search Algorithm (depth = 11, width = 2) has been included to validate the emulator on a deeper quantum circuit.<br>3. BB84 protocol (width = 8) has been added to demonstrate support for a more comprehensive quantum communication protocol.<br>These additions provide validation across both increased circuit width and depth, thereby offering a more thorough assessment of the proposed emulator beyond the initial Bell-state and Deutsch algorithm demonstrations.	Section 4.3,4.4,4.5<br>Section 5-Table 13
2	The emulator is compared with IBM Aer simulator and IBM quantum hardware , but: The comparison is limited to error rates without runtime, latency, or energy efficiency metrics	In response, we have extended the discussion by incorporating runtime comparisons among the proposed FPGA emulator, the IBM Aer simulator, and IBM quantum hardware. A dedicated comparison table and accompanying	Section 5-Table 11

		discussion have been added to highlight the execution speed advantages of the proposed architecture. The revised manuscript now discusses the impact of FPGA parallelism and logic-level execution on reduced runtime.<br><br>In terms of energy efficiency, FPGA-based implementations are generally considered more energy-efficient due to their lower infrastructure and execution overhead compared to large-scale CPU- and QPU-based platforms.	
3	The paper itself acknowledges that hardware noise differences make direct comparison difficult	We thank the reviewer for the observation. The manuscript acknowledges that a direct comparison among FPGA emulation, IBM Aer simulation, and IBM quantum hardware is inherently challenging due to differences in physical hardware noise and execution environments. The intent of the comparison is therefore not to claim exact equivalence, but rather to provide a qualitative reference for validating the functional behavior and output trends of the proposed emulator framework.	–
4	No comparison with other FPGA-based emulators is provided	In response to the valuable feedback, a comprehensive comparison table has been added in the revised manuscript that benchmarks our proposed work against existing state-of-the-art FPGA-based emulators. We believe this addition	Section 5- Table 15

		significantly strengthens the paper and provides readers with a clearer context of our contribution.	

1. Khalid, Madiha, et al. "An FPGA-based hardware abstraction of quantum computing systems." *Journal of Computational Electronics* 20.5 (2021): 2001-2018.
