# Supplementary material for: FPGA-based emulation framework for two-qubit quantum computation: Design, implementation, and validation
Source: PLoS One. 2026 Aug 24;21(8):e0356172. doi: 10.1371/journal.pone.0356172 (PMC13502598; doi:10.1371/journal.pone.0356172)
Supplement: Supplementary file 3 [file pone.0356172.s003.pdf]

Respected editor,

I hope you are fine. Firstly, we would like to thank the anonymous reviewers for their valuable comments and feedback, which will ultimately enhance the paper's overall quality. The following are the reviewers' comments and the actions taken to improve the paper in response to the valuable suggestions.

Comment of Editor:

**S. No.**	**Editor's Comments**	**Author (s) response**	**References**
1	The reviewer still has some concerns regarding the expression. please accordingly revise your MS.	The manuscript has been carefully revised to improve the overall clarity and expression. In addition, several recent and relevant references have been incorporated into the Introduction sections to better contextualize the proposed work.	Section 1: Introduction

Comment of Reviewer:

**S. No.**	**Reviewer's Comments**	**Author (s) response**	**References**
1	The manuscript still contains an inconsistency between the claimed two-qubit emulator and the reported 16-qubit Superdense coding experiment. The title, architecture description, and memory model emphasize a two-qubit framework, while later sections describe a 16-qubit Superdense coding circuit and use it as evidence of scalability.	The manuscript has been revised to clarify the distinction between the underlying two-qubit emulation architecture and the larger validation circuits. The proposed emulator is fundamentally a two-qubit FPGA architecture. The reported 16-qubit Superdense Coding experiment does not represent the simultaneous emulation of a 16-qubit quantum state. Instead, it corresponds to the parallel execution of eight independent two-qubit Superdense Coding circuits, enabling the communication of a 16-bit binary word. The relevant sections have been revised to explicitly clarify this distinction and eliminate any ambiguity regarding the scalability claims.	• Section 1.2: Contributions<br>• Section 4.3: Superdense Coding Circuit<br>• Section 5: Performance Analysis<br>• Table 15

2	The energy-efficiency claim remains insufficiently supported. The revised manuscript discusses runtime and FPGA resource utilization, but the comparison with IBM Aer and IBM hardware does not provide normalized energy, power, or energy-per-operation measurements.	In the revised manuscript, we now report per-gate energy and power for the FPGA and Qiskit Aer (CPU) platforms, derived from Vivado power reports and Intel Power Gadget logs, respectively.<br><br>We acknowledge an inherent asymmetry in measurement methodology between the two platforms.<br>1. FPGA energy/op is obtained through isolated, hardware-level measurements using dedicated logic.<br>2. CPU (Qiskit Aer) energy/op is derived from Intel Power Gadget, which reports total system power and cannot fully isolate a single-gate operation from background OS and process-level activity.<br>Accordingly, we treat the reported CPU values as conservative upper-bound estimates rather than exact intrinsic measurements.<br><br>The inclusion of these measurements in Table 11 is intended to provide perspective on the lightweight and resource-efficient nature of the FPGA implementation rather than to establish a directly comparable energy-efficiency ratio between the heterogeneous platforms.	• Section 5: Performance Analysis<br><br>• Table 11
3	The comparison with IBM quantum hardware is useful but still risks misleading interpretation because physical QPU noise and deterministic FPGA emulation are fundamentally different execution environments.	We agree that FPGA emulation and physical quantum hardware operate under fundamentally different execution environments and therefore do not constitute a direct performance comparison. To avoid any misleading interpretation, the manuscript has been revised to explicitly acknowledge this distinction in the Introduction. The comparison with IBM Quantum Hardware is presented solely for	• Section 1.2: Contributions<br>• Section 5: Performance Analysis

		functional benchmarking and to illustrate the behavior of the proposed emulator relative to widely used quantum computing platforms, rather than to claim equivalent execution conditions or hardware performance.	
4	The fixed-point precision analysis has improved, but the loss of accuracy in deeper circuits remains a limitation. The reported Deutsch and Grover error rates indicate that finite-precision and sampling effects can become non-negligible.	We agree; this is addressed in the manuscript, where the details of emulation error sources describe the four contributing error mechanisms (qubit, gate, multiplication, and measurement error), and the Width and Depth analysis discusses how finite precision and exponential state-space growth affect accuracy in deeper/wider circuits.	Section 5: Performance Analysis
5	Reproducibility remains underdeveloped. For an FPGA-based emulation framework, readers need sufficient access to design artifacts, test benches, circuit descriptions, and simulation settings.	All design artifacts, test benches, circuit descriptions, and simulation settings have been made publicly available and referred (citation number 23,24) as a step-by-step protocol on protocols.io and Github to ensure full reproducibility [1] [2].	Section 3: System Architecture of the Extended FPGA Emulator
6	Some technical and language issues remain, including typographical errors such as "Atrix 7" instead of "Artix 7," inconsistent spacing in terms such as "P hase," and uneven notation for CNOT, QFT, and fixed-point formats. Please conduct a final technical proofreading pass to ensure consistent terminology, notation, figure captions, table labels, and grammar throughout the manuscript.	A thorough technical proofreading of the manuscript has been performed, and typographical errors, notation inconsistencies, formatting, figure captions, table labels, and grammatical issues have been corrected throughout the revised manuscript.	Complete paper

1. https://github.com/Arfeen983/FPGA-Based-Emulation-Framework-for-Two-Qubit-Quantum-Computation.
2. https://dx.doi.org/10.17504/protocols.io.dm6gp4bqpgzp/v2
